# Potential mechanisms of natural products in improving gouty arthritis: Focusing on NLRP3 inflammasome regulation

**DOI:** 10.1016/j.jpha.2026.101563

**Published:** 2026-01-26

**Authors:** Yunhao Yi, Yanling Chen, Wuchaonan Liu, Jingjing Yang, Le Yang, Jing Liu, Shengping Luo, Qianru Zeng, Tao Gao, Yihui Deng

**Affiliations:** a The First Clinical Medical College of Traditional Chinese Medicine, Hunan University of Chinese Medicine, Changsha, 410208, China; b The First Hospital of Hunan University of Chinese Medicine, Hunan University of Chinese Medicine, Changsha, 410021, China; c Hunan Province Key Laboratory of Cerebrovascular Disease Prevention and Treatment of Integrated Traditional Chinese and Western Medicine, Hunan University of Chinese Medicine, Changsha, 410208, China; d College of Integrated Traditional Chinese and Western Medicine, Hunan University of Chinese Medicine, Changsha, 410208, China; e School of Traditional Chinese Medicine, Hunan University of Chinese Medicine, Changsha, 410208, China

**Keywords:** Natural products, Gouty arthritis, NLRP3 inflammasome, Molecular mechanisms, Anti-inflammatory therapy

## Abstract

Gouty arthritis (GA) is a metabolic inflammatory disorder characterized by the deposition of monosodium urate crystals. Although conventional treatments like colchicine can relieve symptoms, they come with several limitations, such as gastrointestinal side effects, hepatorenal toxicity, and poor target specificity. In contrast, natural products, known for their multi-pathway and multi-target properties, present distinct advantages and significant potential for intervening in GA. The primary pathological mechanism of GA involves the overactivation of the NOD-like receptor family pyrin domain-containing 3 (NLRP3) inflammasome, making its regulation a promising therapeutic approach. However, there is currently a lack of comprehensive reviews on how natural products target the NLRP3 inflammasome. Additionally, existing studies have not sufficiently classified or integrated the mechanisms involved in transcriptional and translational regulation during the priming phase, post-translational modifications, and the various pathways active during the activation phase. This article aims to address these gaps through a systematic literature review, providing an in-depth analysis of the molecular mechanisms by which natural products alleviate GA by targeting the NLRP3 inflammasome. It also critically examines the major challenges in current translational research, with the objective of offering a solid theoretical foundation and practical strategies for developing novel, effective anti-inflammatory drugs and expediting the clinical application of natural products for GA treatment.

## Highlights

• Multiple mechanisms contribute to NLRP3 inflammasome overactivation, which drives the pathogenesis of GA.
• Natural products regulate GA by modulating priming signals, post-translational modifications, and inflammasome activation.
• Natural products directly target inflammasome structural domains or protein-protein interactions, inhibiting assembly.
• Natural products offer multi-target synergy, but toxicity, low bioavailability, and poor targeted delivery remain challenges.
• Elucidating how natural products regulate the NLRP3 inflammasome in GA will advance translational research.

## Graphical abstract

**Figure:**
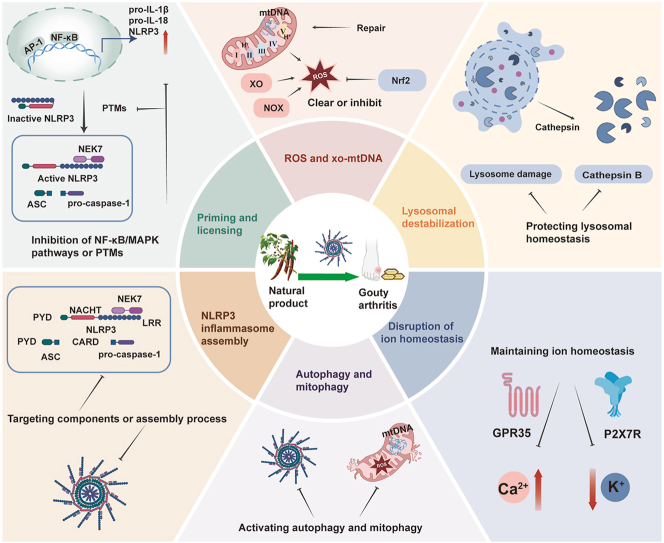

## 1 Introduction

Gouty arthritis (GA) is an inflammatory condition that occurs due to the deposition of monosodium urate (MSU) crystals in joint capsules, bursae, cartilage, and other tissues [1]. Acute GA attacks are marked by intense "knife-like" or "gnawing" pain, accompanied by joint redness, swelling, warmth, and functional impairment. These recurrent episodes can lead to chronic arthritis, which in turn may cause joint deformity and limited mobility. In 2021, it was estimated that gout affected approximately 56.47 million individuals worldwide [2], and projections suggest this number will rise to 95.8 million by 2050 [3]. This indicates a steadily increasing prevalence worldwide, particularly in developed nations. Pathophysiologically, the accumulation of MSU crystals in and around the joints activates the immune system, initiating an inflammatory cascade. This cascade results in the substantial release of pro-inflammatory cytokines, such as interleukin-1beta (IL-1β), which further intensifies local inflammation [4]. Current first-line treatments for gout attacks, such as colchicine and nonsteroidal anti-inflammatory drugs, focus on symptom relief. However, these treatments can lead to adverse effects, including gastrointestinal injury, hepatorenal toxicity, and an increased risk of cardiovascular disease with long-term use. Despite employing conventional urate-lowering and anti-inflammatory strategies, gout attacks often recur. This highlights the urgent need to clarify the inflammatory mechanisms underlying GA and to develop more effective and safer therapeutic agents.

In recent years, natural products have garnered attention for their potential to modulate multiple pathways and their low toxicity [5]. These compounds, including alkaloids, flavonoids, terpenoids, and polysaccharides, are primary or secondary metabolites derived from natural sources such as plants, microorganisms, and marine organisms. Compared to conventional synthetic drugs, natural products have unique multi-target capabilities, making them promising candidates for therapeutic development [6]. Their diverse chemical structures serve as a valuable resource for identifying novel bioactive molecules that target key mechanisms in GA pathogenesis. Unlike single-target agents, natural products can modulate multiple pathological processes involved in GA simultaneously, potentially resulting in synergistic effects. Furthermore, these natural compounds generally exhibit higher biocompatibility and fewer adverse effects, such as hepatotoxicity, nephrotoxicity, or allergic reactions, compared to synthetic alternatives.

A critical mediator of innate immune responses, the NOD-like receptor family pyrin domain-containing 3 (NLRP3) inflammasome, plays a significant role in the development of GA. The activation of this complex occurs through a two-step process: priming and triggering. However, the precise mechanisms by which MSU stimulation activates the NLRP3 inflammasome remain incompletely defined, particularly regarding ion flux disturbances, mitochondrial dysfunction, and specific post-translational modifications (PTMs), etc. [7]. Several studies have highlighted the therapeutic potential of targeting the NLRP3 inflammasome with natural products for the treatment of GA. For example, phenolic compounds, flavonoids, and alkaloids derived from traditional Chinese medicine, such as carvacrol and berberine, have been shown to reduce MSU crystal-induced inflammation by suppressing nuclear factor kappa-light-chain-enhancer of activated B cells (NF-κB) signaling or inhibiting NLRP3 inflammasome assembly [8]. NLRP3 inflammasome-targeted strategies utilizing natural products present a promising approach to address the limitations of current GA therapies. However, existing research falls short in providing a systematic classification of the mechanisms involved and a comprehensive summary of relevant natural products.

This paper aims to elucidate the molecular mechanisms through which the NLRP3 inflammasome mediates inflammatory responses in GA. Additionally, we will systematically summarize and categorize natural products that target the NLRP3 inflammasome for GA treatment, establishing a scientific foundation for future basic research and clinical applications.

## 2 Review methodology

**Fig. 1:**
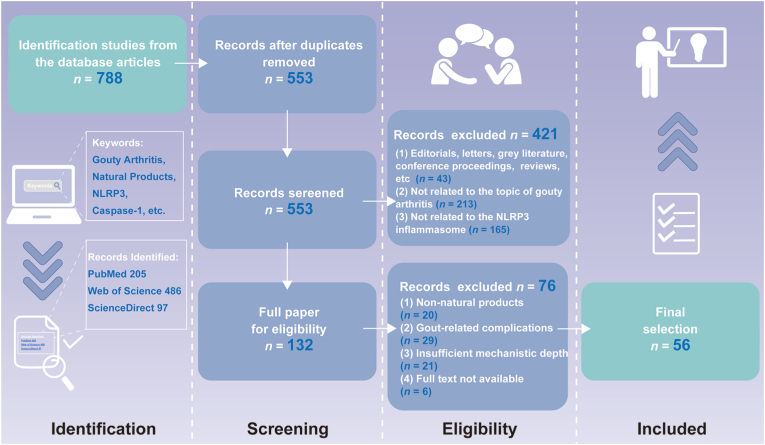
Retrieval process.

To clarify the mechanistic basis of how natural products alleviate GA through the modulation of the NLRP3 inflammasome pathway, a systematic literature review was conducted in accordance with the Preferred Reporting Items for Systematic Reviews and Meta-Analyses (PRISMA) statement guidelines. The search was carried out in PubMed, Web of Science, and ScienceDirect databases. Keywords used in the search included: "natural products", "gout", "gouty arthritis", "NLRP3 inflammasome", "IL-1β", "interleukin-18 (IL-18)", "caspase-1", "pyroptosis", and "monosodium urate". Two reviewers independently screened the retrieved literature based on the title, abstract, and full text, adhering strictly to the inclusion and exclusion criteria. The inclusion criteria were: (1) original research articles published in English; and (2) studies that investigate the role of natural products in treating GA by regulating the NLRP3 inflammasome in gout-related models. The exclusion criteria included: (1) non-English literature; (2) grey literature; (3) opinion pieces; (4) review articles; and (5) duplicate publications. After the literature search and screening process, a total of 56 articles on the treatment of GA with natural products focusing on the NLRP3 inflammasome were included in this study. The retrieval process is shown in Fig. 1.

## 3 Overview of the NLRP3 inflammasome

Inflammation is a defense response triggered by pathogen infection or tissue damage. As a critical signaling hub in this process, inflammasomes intricately connect the recognition of danger signals to the activation of downstream defensive responses. Among these, the NLRP3 inflammasome has been the most extensively studied in mammals and is regarded as a promising candidate for drug targeting [9]. The deeper understanding of the NLRP3 inflammasome's structure, activation mechanisms, and regulatory networks is essential for developing targeted anti-inflammatory therapeutics.

### 3.1 Structure and function of the NLRP3 inflammasome

#### 3.1.1 NLRP3

NOD-like receptors (NLRs) are a class of cytoplasmic pattern recognition receptors (PRRs). The NLRP subfamily is the largest among NLRs, with NLRP3 being the most extensively studied member. The NLRP3 protein consists of three domains: an N-terminal pyrin domain (PYD), a central nucleotide-binding domain (NACHT), and a C-terminal leucine-rich repeat (LRR) region. The N-terminal PYD mediates homotypic PYD-PYD interactions and recruits the adaptor protein apoptosis-associated speck-like protein containing a caspase recruitment domain (ASC). The C-terminal LRR domain maintains autoinhibition in the resting state by occluding the NACHT domain. It also senses various activation signals and facilitates inflammasome assembly through PTMs and interaction with NIMA-related kinase 7 (NEK7) [10]. The central NACHT domain functions as the core effector module of NLRP3. It integrates upstream signals and facilitates adenosine triphosphate (ATP)-dependent oligomerization [11].

#### 3.1.2 ASC

The adaptor protein ASC contains an N-terminal PYD and a C-terminal caspase activation and recruitment domain (CARD). The N-terminal PYD interacts homotypically with the PYD of NLRP3, while the C-terminal CARD domain binds the downstream effector protein pro-caspase-1 [12]. Upon NLRP3 activation and oligomerization, ASC is recruited through PYD-PYD interactions, which trigger its self-oligomerization into large, speck-like aggregates known as ASC specks. The formation of these ASC specks serves as a key morphological indicator of inflammasome activation and signals the onset of pyroptosis [13]. These structures efficiently recruit multiple pro-caspase-1 molecules through CARD-CARD interactions, thereby amplifying the inflammatory signal. Given its central role, ASC and its oligomerization represent promising targets for anti-inflammatory therapies.

#### 3.1.3 Pro-caspase-1

Pro-caspase-1 is composed of an N-terminal CARD domain, a central large subunit (p20), and a C-terminal small subunit (p10). In its inactive form, pro-caspase-1 exists as either a monomer or a dimer, lacking catalytic activity. The recruitment of pro-caspase-1 into ASC specks induces its clustering, resulting in a high local concentration that facilitates homodimerization. This process is followed by autoproteolytic cleavage and structural rearrangement, ultimately leading to the formation of the active caspase-1 heterotetramer [14]. Active caspase-1 cleaves precursor interleukin-1 beta (pro-IL-1β), pro-IL-18, and gasdermin D (GSDMD), thereby mediating cytokine maturation and pyroptotic cell death [15].

### 3.2 Classical pathway of NLRP3 inflammasome activation

#### 3.2.1 Priming

The priming step provides the molecular prerequisites for NLRP3 inflammasome activation by relieving NLRP3 autoinhibition and upregulating inflammasome components. The binding of damage-associated molecular patterns (DAMPs), pathogen-associated molecular patterns (PAMPs), or proinflammatory cytokines to their specific receptors triggers downstream signaling cascades that converge on the activation of NF-κB. Once activated, the NF-κB dimer translocates to the nucleus, where it drives the transcriptional upregulation of NLRP3, pro-IL-1β, and pro-IL-18.

A canonical example of this process is the binding of lipopolysaccharide (LPS) to Toll-like receptor 4 (TLR4). This interaction initiates a myeloid differentiation primary response 88 (MyD88)-dependent signaling cascade, which activates the IκB kinase (IKK) complex. The IKK complex then phosphorylates the inhibitory protein inhibitor of κB alpha (IκBα), marking it for degradation. This degradation releases NF-κB, allowing it to translocate to the nucleus and initiate the transcription of proinflammatory genes [16]. The mitogen-activated protein kinase (MAPK) pathway can also be activated and cooperate with NF-κB to enhance the expression of these genes [17].

Priming signals alone are not enough to trigger inflammasome assembly or caspase-1 activation. However, they do prepare the cell for a swift response by ensuring sufficient levels of NLRP3 and pro-cytokines. Recent studies have identified NF-κB/MAPK-independent mechanisms that regulate NLRP3 transcription. For instance, in an LPS-induced septic shock model, S-nitrosoglutathione reductase suppresses NLRP3 expression by limiting MAPK14 S-nitrosylation [18]. A deeper understanding of alternative transcriptional regulatory mechanisms will enhance the development of targeted therapeutic strategies for precisely intervening in NLRP3 inflammasome-mediated inflammatory diseases.

#### 3.2.2 Activation

Current evidence shows that NLRP3 inflammasome activation can be triggered by PAMPs and DAMPs, leading to upstream signaling events such as the overproduction of reactive oxygen species (ROS) lysosomal disruption, mitochondrial dysfunction, and disturbances in ion homeostasis. As research has advanced, it has become evident that NLRP3 inflammasome activation involves not only the detection of disruptions in cellular homeostasis but also complex regulation through PTMs and various regulatory factors [19]. Nevertheless, the precise mechanisms by which these signals promote NLRP3 inflammasome assembly are still not fully understood. It is still a matter of debate whether these pathways operate independently or interact with each other under specific conditions [7].

In its resting state, NLRP3 remains in an autoinhibited oligomeric conformation. Upon cellular stress, the LRR domain senses homeostatic disturbances and relieves NACHT-mediated autoinhibition. The kinase NEK7 serves as a key positive regulator by binding to NLRP3 and destabilizing its inactive structure [20]. This interaction induces a conformational shift that allows multiple NLRP3 molecules to assemble into rotor-like oligomers through NACHT-NACHT associations. Oligomerization exposes the N-terminal PYD, enabling recruitment of the adaptor ASC through homotypic PYD-PYD engagement, thereby initiating prion-like ASC speck formation [21]. The exposed ASC CARD then associates with the CARD of pro-caspase-1, promoting its autoproteolytic activation. Active caspase-1 processes pro-IL-1β and pro-IL-18 into their mature cytokines and cleaves GSDMD at its interdomain linker [22]. he liberated N-terminal fragment of GSDMD (GSDMD-N) fragments oligomerize to form membrane pores, driving pyroptosis, a lytic cell-death program characterized by the release of inflammatory mediators.

Further investigations have revealed that the activation of the NLRP3 inflammasome is subject to precise spatiotemporal regulation. Its assembly is not random; instead, it is coordinated at specific organellar sites and occurs in a temporally controlled sequence [23]: from the dissolution of the autoinhibited NLRP3 structure, to NEK7-mediated discoid oligomer formation, and finally to ASC recruitment and caspase-1 activation [7].

**Fig. 2:**
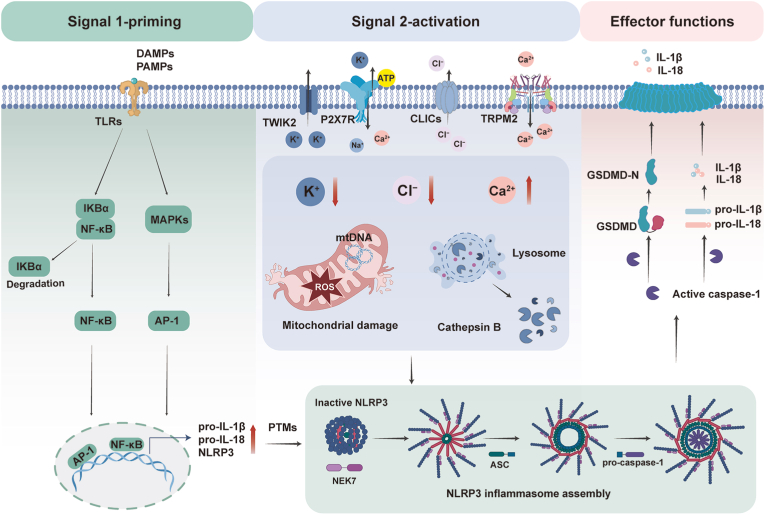
The priming and activation of the NOD-like receptor family pyrin domain containing 3 (NLRP3) inflammasome. AP-1: activator protein 1; ASC: apoptosis-associated speck-like protein containing a caspase recruitment domain; ATP: adenosine triphosphate; CLICs: chloride intracellular channels; DAMPs: damage-associated molecular patterns; GSDMD: gasdermin D; GSDMD-N: N-terminal fragment of gasdermin D; IκBα: inhibitor of NF-κB alpha; IL-1β: interleukin-1 beta; IL-18: interleukin-18; MAPKs: mitogen-activated protein kinases; mtDNA: mitochondrial DNA; NEK7: NIMA-related kinase 7; NF-κB: nuclear factor kappa B; NLRP3: NOD-like receptor family pyrin domain containing 3; P2X7R: P2X7 receptor; PAMPs: pathogen-associated molecular patterns; pro-IL-1β: pro-interleukin-1 beta; pro-IL-18: pro-interleukin-18; PTMs: post-translational modifications; ROS: reactive oxygen species; TLRs: Toll-like receptors; TRPM2: transient receptor potential melastatin 2; TWIK2: tandem of pore domains in a weak inward rectifying K^+^ channel 2.

NLRP3 activation involves critical PTMs that serve as permissive signals for inflammasome activation. These PTMs can prevent overactivation while also promoting the assembly of the NLRP3 inflammasome, creating a complex regulatory network. Key PTMs include phosphorylation and dephosphorylation, ubiquitination and deubiquitination, and acetylation and deacetylation, among others. Despite their importance, targeting PTMs remains uncertain and challenging. Notably, several drugs have been identified that specifically target these modifications, such as the phosphorylation inhibitor Bruton's tyrosine kinase inhibitor, the deubiquitination inhibitor holomycin, and the palmitoylation inhibitor disulphalan [24]. PTMs can dynamically modify components of the NLRP3 inflammasome, presenting a promising strategy for treating NLRP3 inflammasome-related inflammatory diseases. In summary, the regulation of the NLRP3 inflammasome involves numerous mechanisms, creating a complex network. Future research is necessary to fully integrate these molecular regulatory pathways. The activation mechanism of the NLRP3 inflammasome, including its priming and subsequent activation phases, is illustrated in Fig. 2.

### 3.3 Non-classical pathways and alternative pathways

The non-classical activation pathway plays a crucial role in detecting and eliminating Gram-negative bacteria that invade the host cytoplasm. When these bacteria breach the host membrane structure using secretion systems or pore-forming toxins, the cell wall component LPS is released as a cytoplasmic danger signal. Importantly, LPS is recognized directly by the cytosolic machinery, rather than being sensed through membrane receptors. This recognition is mediated by caspase-11 in mice and its homologs, caspase-4 and caspase-5, in humans. Once LPS binds to the CARD domain of these caspases, it triggers their self-activation. Subsequently, activated caspase-11/4/5 cleaves the GSDMD protein, leading to pyroptosis, instead of cleaving the precursors of IL-1β and IL-18 directly [25]. The GSDMD-mediated membrane pores cause intracellular potassium ion efflux, which can serve as a second signal to activate the classical NLRP3 inflammasome, ultimately promoting the maturation and release of IL-1β and IL-18 [26]. However, the regulatory differences in this pathway between humans and mice, as well as its application in disease models, still require verification, particularly in the context of chronic inflammation and tissue damage.

The alternative NLRP3 inflammasome pathway is a newly discovered pro-inflammatory mechanism that functions independently of the classical NLRP3 inflammasome activation pathway. This pathway has primarily been studied in human monocytes [27]. Its key feature is that it does not rely on potassium ion efflux, cell pyroptosis, or classical inflammasome activators (such as ATP or nigericin) [28]. In this process, LPS activates NLRP3 inflammasome and caspase-1 via the TLR4–TIR-domain-containing adapter-inducing interferon-β (TRIF)–receptor-interacting serine/threonine-protein kinase 1 (RIPK1)–Fas-associated death domain protein (FADD)–caspase-8 signaling axis. This activation leads to the maturation and secretion of IL-1β without requiring an additional second signal.

## 4 Molecular regulation of the NLRP3 inflammasome in GA

### 4.1 Priming phase

#### 4.1.1 NF-κB signaling pathway

The TLRs/NF-κB signaling pathway is a major upstream regulatory mechanism that initiates NLRP3 inflammasome activation, and its role in GA has been supported by both preclinical and clinical studies. Clinical data show that the messenger ribonucleic acid (mRNA) and protein levels of TLR4, p65 subunit of NF-κB (p65), and caspase-1 are significantly elevated in peripheral blood mononuclear cells (PBMCs) from patients with acute GA [29]. Genetic studies have further demonstrated that the T allele of the TLR4 gene rs2149356 polymorphism increases the risk of GA in both European and Chinese populations [30,31]. In a rodent model of MSU crystal-induced GA, there was a significant upregulation of TLRs, MyD88, and phosphorylated p65 proteins in the synovial tissues [32].

Mechanistic studies have shown that TLR2 and TLR4 recognize MSU crystals and upregulate transcriptional upregulation of inactive NLRP3, pro-IL-1β, and pro-IL-18 through activation of NF-κB signaling [33]. Gene knockout experiments further confirmed this, revealing that bone marrow-derived macrophages lacking TLR2 or TLR4 exhibited significantly reduced recognition and response to MSU crystals [34]. In addition to TLRs, adhesion molecules are also believed to be involved in the regulation of this signaling pathway. Cluster of differentiation 44 (CD44) serves as a key receptor for MSU crystal uptake, promoting GA inflammation by coordinating phagocytosis, NF-κB signaling, and NLRP3 inflammasome activation [35].

The activation of NF-κB signaling depends on the phosphorylation regulation of multiple key signaling proteins. For instance, inhibiting transforming growth factor-beta-activated kinase 1 (TAK1) phosphorylation suppresses NF-κB activation, and limits NLRP3 inflammasome assembly, ultimately mitigating GA associated inflammation [36,37]. In addition, dynamin-related protein 1 (Drp1) knockdown suppresses mitochondrial fission and ROS generation, thereby blocking the phosphorylation of p65 and IκBα and ultimately reducing inflammation in GA [38].

Recent studies have highlighted the critical role of epigenetic factors in regulating this pathway. Among these, bromodomain-containing protein 4 (BRD4) stands out as a key epigenetic regulatory factor and is regarded as a promising therapeutic target for GA [39]. Inhibition of BRD4 can block its mediation of nuclear p65 phosphorylation, thereby suppressing NLRP3 inflammasome activation and alleviating acute gout attacks induced by MSU [40]. Notably, IL-1β and tumor necrosis factor alpha (TNF-α) released downstream of the NLRP3 inflammasome pathway further activate the priming stage through their receptors, forming a positive feedback loop that amplifies the inflammatory response [41]. These findings collectively establish the NF-κB pathway as a central regulatory hub that connects the inflammatory potential of MSU crystals to the activation of the NLRP3 inflammasome. This identification highlights the NF-κB pathway as a promising therapeutic target for GA.

#### 4.1.2 MAPK signaling pathway

MAPK signaling pathways, including extracellular signal-regulated kinase (ERK), c-Jun N-terminal kinase (JNK), and p38 mitogen-activated protein kinase (p38 MAPK), work in conjunction with NF-κB to activate NLRP3 inflammasomes. In GA, MSU crystals specifically activate the p38 MAPK signaling pathway, which subsequently regulates the translational synthesis of pro-IL-1β [42]. Studies have indicated that the regulation of translation initiation involves both mitogen-activated protein kinase-interacting kinase 1 (Mnk1) and mitogen-activated protein kinase-interacting kinase 2 (Mnk2), which are downstream effectors of p38. Their mechanisms are closely associated with stabilizing pro-IL-1β mRNA. Further research has revealed that Mnk1 and Mnk2 achieve this stabilization by inhibiting the mRNA-destabilizing factor tristetraprolin (TTP) [43]. Targeted modulation of TTP function has been shown to influence inflammatory progression. For instance, protein phosphatase 2A (PP2A) agonists significantly mitigate MSU crystal-induced GA by enhancing TTP-mediated mRNA degradation [44]. Additionally, targeting and inhibiting the phosphorylation or expression of key nodes like p38, JNK, or ERK1 can work synergistically to suppress NLRP3 inflammasome activation, ultimately alleviating inflammation in GA [45]. Current research on the role of the MAPK pathway in GA is still limited, especially regarding PTMs. However, these findings highlight the significant therapeutic potential of targeting the MAPK pathway for developing new treatment strategies for GA.

### 4.2 Activation phase

#### 4.2.1 ROS accumulation and NLRP3 inflammasome activation

ROS facilitates the assembly of the NLRP3 inflammasome through several mechanisms. These include direct oxidative modification, the release of DAMPs, disruption of organelle homeostasis, and the modulation of inflammatory signaling pathways. In clinical studies, patients with GA consistently show elevated systemic oxidative stress and related joint damage. This is evidenced by increased circulating levels of oxidative stress biomarkers, such as malondialdehyde, as well as inflammatory markers like C-reactive protein [46,47].

In experimental GA models, MSU crystals activate nicotinamide adenine dinucleotide phosphate (NADPH) oxidase (NOX) and impair mitochondrial membrane function, resulting in ROS production and subsequent NLRP3 inflammasome activation [48]. Mitochondrial ROS (mtROS) represents a central ROS source for NLRP3 inflammasome activation, with their generation tightly linked to disturbances in Ca^2+^ homeostasis [49]. Recent studies indicate that MSU can cause excessive Ca^2+^ entry into mitochondria or abnormal transfer from the endoplasmic reticulum to mitochondria. This leads to mitochondrial Ca^2+^ overload and dysfunction of the electron transport chain (ETC), ultimately resulting in the massive production of mtROS. This overload may trigger the opening of the mitochondrial permeability transition pore (mPTP), leading to the release of mtROS and mitochondrial deoxyribonucleic acid (mtDNA), which directly activate the NLRP3 inflammasome [50,51]. Notably, xanthine oxidase (XO), an enzyme involved in uric acid synthesis, is also an important source of ROS, and its pharmacological inhibition can significantly reduce ROS levels and alleviate inflammatory responses [52].

The serine/threonine phosphatase PP2A plays a crucial role in regulating cellular reduction-oxidation homeostasis. Research indicates that PP2A alleviates acute gouty inflammation by inhibiting the XO/ROS signaling pathway, which reduces NLRP3 inflammasome activation. However, an excessive accumulation of ROS can suppress PP2A activity, creating a pro-inflammatory positive feedback loop [53]. The nuclear factor erythroid 2-related factor 2 (Nrf2) pathway protects against the accumulation of ROS and the activation of the NLRP3 inflammasome by promoting the expression of antioxidant enzymes [54]. In pathological conditions, enhancing Nrf2 reduces ROS accumulation, suppresses NLRP3 inflammasome activation, and alleviates GA symptoms.

ROS induces dissociation of thioredoxin (TRX) from thioredoxin-interacting protein (TXNIP), allowing TXNIP to activate the NLRP3 inflammasome [55]. In addition, ROS in the GA model also promotes IL-1β release by activating NF-κB signaling during the priming phase, primarily through phosphorylation and degradation of IκBα [56]. In conclusion, the accumulation of ROS not only initiates NLRP3 inflammasome-mediated inflammatory cascades but also exacerbates oxidative stress, which in turn exacerbates joint damage. These findings emphasize the importance of monitoring ROS dynamics and highlight the therapeutic potential of targeting ROS-regulating pathways in the management of GA.

#### 4.2.2 mtDNA release and NLRP3 inflammasome activation

In human cells, mtDNA is the genetic material found within mitochondria. This circular molecule is not associated with histones, which imparts unique biological properties. Preliminary studies indicate that mtDNA heteroplasmy occurs more frequently in GA patients, suggesting a close association between mitochondrial genomic instability and the development of the disease [57]. Further research has identified mtDNA variations as an independent genetic risk factor for GA, potentially contributing to disease pathogenesis through disruption of mitochondrial functions, including energy metabolism, oxidative stress regulation, and inflammatory signaling [58].

mtDNA is highly susceptible to oxidation by ROS, leading to the formation of oxidized mtDNA (ox-mtDNA). This oxidized form acts as a DAMP that is recognized by the NLRP3, initiating the assembly of the inflammasome [59]. Studies have confirmed that extracellular mtDNA released by MSU crystals activates the TLR9-dependent NF-κB signaling pathway. This activation promotes the transcription and translation of inflammatory proteins associated with this pathway [60]. GSDMD-mediated pyroptotic pores increase the release of mtDNA, which in turn amplifies the inflammatory response in GA. In summary, mtDNA is not only related to the genetic susceptibility of GA but also can participate in the pathological process of GA as a DAMP.

#### 4.2.3 Perturbation of lysosomal homeostasis and activation of NLRP3 inflammasome

Numerous studies have shown that lysosomal damage signals can be sensed by NLRP3, leading to activation of the NLRP3 inflammasome. In GA, upon phagocytosis of MSU crystals by macrophages, lysosomal membrane permeabilization occurs, leading to membrane rupture and the release of cathepsin B into the cytoplasm [61]. Preclinical studies have shown that, during MSU or LPS stimulation, the absence of cathepsin B significantly suppresses caspase-1 activation and IL-1β production in bone marrow-derived macrophages. Importantly, this effect occurs without impacting NLRP3 expression, highlighting cathepsin B as a critical mediator of inflammasome activation [62]. Further research has found that cathepsin B released by the unstable rupture of lysosomes caused by MSU can be recognized by NLRP3, promoting the assembly and activation of inflammasomes [63]. Animal studies further support this mechanism by demonstrating that blocking the interaction between cathepsin B and NLRP3 effectively suppresses inflammasome activation. This inhibition also alleviates MSU crystal-induced GA symptoms in mice [61].

In addition to signal perception, lysosomes also play a key scaffolding role in the spatial assembly of NLRP3 inflammasomes. In GA models, the lysosome membrane-anchored ragulator complex (with Lamtor1 as the core component) can bind to histone deacetylase 6 and provide a spatial scaffold to promote the transport and aggregation of NLRP3 to the microtubule-organizing center (MTOC) [64]. This allows NLRP3 to aggregate in the MTOC region and interact with the adaptor protein ASC, which promotes the assembly and activation of the inflammasome.

In summary, lysosomes play a crucial role in the activation of the NLRP3 inflammasome in GA by altering membrane permeability, releasing cathepsin B, and serving a spatial scaffold function. Further exploration of its role as a spatial scaffold may reveal new targets and strategies for treating GA.

#### 4.2.4 Disturbance of intracellular ion homeostasis and NLRP3 inflammasome activation

Disruption of intracellular ion homeostasis, including K^+^ efflux, Ca^2+^ influx, and Cl^−^ efflux, represents a critical upstream signal for NLRP3 inflammasome activation. While direct studies connecting ion homeostasis disorders to NLRP3 inflammasome activation in GA patients are still limited, there is growing evidence indicating that this mechanism is crucial in the pathogenesis of GA. Current evidence highlights tandem of pore domains in a weak inward rectifying K^+^ channel 2 (TWIK2)-mediated K^+^ efflux as a pivotal upstream event in MSU crystal-induced NLRP3 inflammasome activation [65]. Another study demonstrated that MSU crystals release Na^+^ in the acidic lysosomal environment. This release increases intracellular osmotic pressure, leading to water influx through aquaporins. As a result, intracellular K^+^ is diluted, which activates the NLRP3 inflammasome and promotes IL-1β maturation [66]. Chloride intracellular channels (CLICs)-dependent Cl^−^ efflux has also been identified as a crucial upstream signal for NLRP3 inflammasome activation [67]. K^+^ efflux induced by MSU crystals can cause mitochondrial damage and increase the production of mtROS. This process promotes the translocation of CLICs channels to the plasma membrane, which mediates Cl^−^ efflux and activates the inflammasome. Additionally, K^+^ efflux directly enhances the interaction between NEK7 and NLRP3, facilitating the assembly of the inflammasome.

The P2X7 receptor (P2X7R), which mediates K^+^ efflux as well as Ca^2+^ and Na^+^ influx, is considered a promising therapeutic target for GA [68]. Studies have demonstrated that ATP-induced disruption of ion homeostasis via P2X7R enhances NLRP3 inflammasome activation and IL-1β release, thereby driving the progression of GA [69,70]. Calcium signaling and NLRP3 inflammasome activation are further interconnected through mitochondrial dysfunction [71]. Transient receptor potential melastatin 2 channels have been shown to mediate Ca^2+^ influx in response to MSU crystal stimulation [72]. Moreover, MSU crystals can trigger the release of Ca^2+^ from the endoplasmic reticulum into the mitochondria. This process leads to mitochondrial calcium overload, which disrupts the membrane potential, causes overproduction of mtROS, and results in the release of mtDNA [73]. This Ca^2+^ transfer is primarily mediated by the inositol 1,4,5-trisphosphate receptor (IP3R) on the endoplasmic reticulum and the mitochondrial calcium uniporter (MCU)/voltage-dependent anion channel (VDAC) on mitochondria [50,73,74]. In summary, growing evidence indicates that disturbances in intracellular ion homeostasis serve as key upstream signals for NLRP3 inflammasome activation in GA models. However, the exact mechanisms of ion signal crosstalk and their dynamic interactions with organelles like mitochondria and the endoplasmic reticulum have yet to be fully elucidated.

#### 4.2.5 Autophagy and mitophagy exert inhibitory regulation on the NLRP3 inflammasome

Autophagy is a crucial process for maintaining cellular homeostasis and balancing inflammation. It plays a key role in regulating NLRP3 inflammasome activation and inflammatory responses in GA. Clinical studies have demonstrated that PBMCs from GA patients exhibit reduced expression of autophagy-related proteins microtubule-associated protein 1 light chain 3 (LC3) and Beclin-1 following exposure to MSU crystals, which is negatively correlated with serum IL-1β levels [75]. Animal model studies further support the protective role of autophagy. For instance, autophagy inducers like rapamycin exhibit significant anti-inflammatory effects, underscoring autophagy's critical involvement in regulating MSU crystal-induced GA [76]. However, the precise mechanisms underlying this regulation in GA remain incompletely understood.

Preclinical evidence shows that in MSU crystal-treated THP-1 cells, LC3-II and Beclin-1 levels are markedly decreased, while sequestosome-1 (p62) accumulates, suggesting impaired autophagic activity [77]. Further mechanistic studies reveal that MSU crystals inhibit the phosphorylation of AMP-activated protein kinase alpha (AMPKα) at Thr172, which leads to the inactivation of the AMPK complex. This inactivation disrupts autophagic flux and prevents the suppression of NLRP3 inflammasome activation [78]. Recent studies indicate that the overexpression of dual specificity phosphatase 1 enhances autophagy, facilitates the clearance of mitochondrial damage caused by MSU crystals, reduces mtROS release, and consequently suppresses the assembly of the NLRP3 inflammasome [79]. Sirtuin 1 (SIRT1) is a nicotinamide adenine dinucleotide (NAD^+^)-dependent deacetylase that often functions as a "regulatory switch" in cells. In PBMCs from GA patients, MSU crystal stimulation inhibits SIRT1 expression, leading to increased p65 acetylation that drives the transcription of the NLRP3 inflammasome and induces GA inflammation. Conversely, the activation of SIRT1 enhances Beclin-1/LC3-II-mediated autophagy, which promotes the ubiquitination and autophagic degradation of NLRP3, thereby alleviating GA inflammation [75]. MSU crystals also impair mitophagy, leading to the accumulation of dysfunctional mitochondria and reactive mtROS, which continuously activate the NLRP3 inflammasome [80]. Notably, activated caspase-1 can cleave and inactivate Parkin, thereby suppressing PTEN-induced kinase 1 (Pink1)/Parkin-mediated mitophagy [81].

**Fig. 3:**
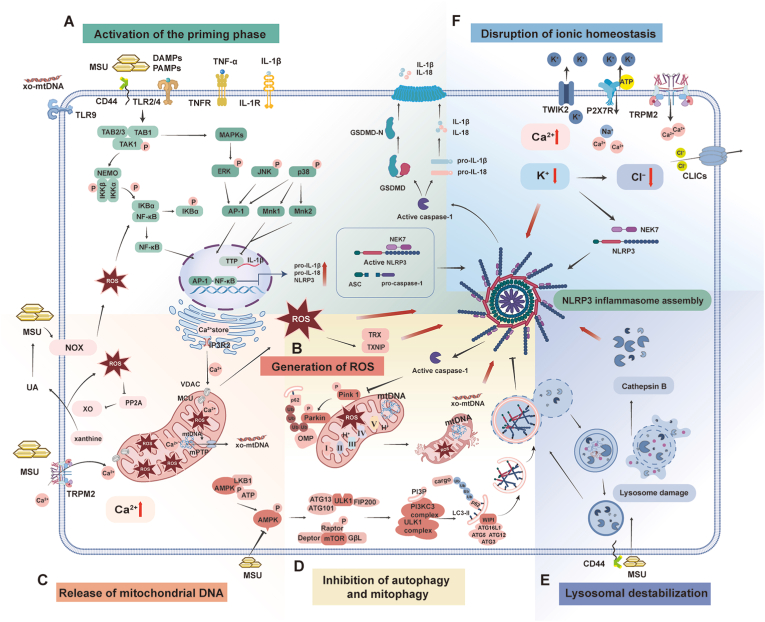
Priming and activation of the NOD-like receptor family pyrin domain containing 3 (NLRP3) inflammasome in gouty arthritis. (A) Activation of the priming phase. (B) Generation of reactive oxygen species (ROS). (C) Release of mitochondrial DNA. (D) Inhibition of autophagy and mitophagy. (E) Lysosomal destabilization. (F) Disruption of ionic homeostasis. AMPK: AMP-activated protein kinase; AP-1: activator protein 1; ASC: apoptosis-associated speck-like protein containing a caspase recruitment domain; ATG3: autophagy-related 3; ATG5: autophagy-related 5; ATG12: autophagy-related 12; ATG13: autophagy-related 13; ATG16L1: autophagy-related 16-like 1; ATG101: autophagy-related 101; ATP: adenosine triphosphate; CD44: cluster of differentiation 44; CLICs: chloride intracellular channels; DAMPs: damage-associated molecular patterns; Deptor: DEP domain-containing mTOR-interacting protein; ERK: extracellular signal-regulated kinase; FIP200: FAK family kinase-interacting protein of 200 kDa; GβL: G protein beta subunit-like (mLST8); GSDMD: gasdermin D; GSDMD-N: N-terminal fragment of gasdermin D; IKKα: IκB kinase alpha; IKKβ: IκB kinase beta; IKKγ (NEMO): IκB kinase gamma; IL-1β: interleukin-1 beta; IL-1R: interleukin-1 receptor; IL-18: interleukin-1 beta; IP3R2: inositol 1,4,5-trisphosphate receptor type 2; JNK: c-Jun N-terminal kinase; LKB1: liver kinase B1; LC3-II: lipidated microtubule-associated protein 1 light chain 3; MAPKs: mitogen-activated protein kinases; MCU: mitochondrial calcium uniporter; Mnk1: mitogen-activated protein kinase-interacting kinase 1; Mnk2: mitogen-activated protein kinase-interacting kinase 2; mPTP: mitochondrial permeability transition pore; MSU: monosodium urate; mtDNA: mitochondrial DNA; mTOR: Mechanistic target of rapamycin; NEK7: NIMA-related kinase 7; NF-κB: nuclear factor kappa B; NLRP3: NOD-like receptor family pyrin domain containing 3; NOX: NADPH oxidase; OMP: outer mitochondrial membrane (proteins); ox-mtDNA: oxidized mitochondrial DNA; P2X7R: P2X7 receptor; p38: p38 mitogen-activated protein kinase; p62: sequestosome 1; PAMPs: pathogen-associated molecular patterns; PI3KC3: class III phosphatidylinositol 3-kinase; PI3P: phosphatidylinositol 3-phosphate; PP2A: protein phosphatase 2A; Raptor: regulatory-associated protein of mTOR; ROS: reactive oxygen species; TAB1: TAK1-binding protein 1; TAB2/3: TAK1-binding protein 2/3; TAK1: transforming growth factor-beta-activated kinase 1; TLR2/4: Toll-like receptor 2/4; TLR9: Toll-like receptor 9; TNF-α: tumor necrosis factor alpha; TNFR: tumor necrosis factor receptor; TRPM2: transient receptor potential melastatin 2; TRX: thioredoxin; TTP: tristetraprolin; TWIK2: tandem of pore domains in a weak inward rectifying K^+^ channel 2; TXNIP: thioredoxin-interacting protein; UA: uric acid; Ub: ubiquitin; ULK1: Unc-51-like kinase 1; VDAC: voltage-dependent anion channel; WIPI: WD repeat domain phosphoinositide-interacting protein; XO: xanthine oxidase.

In summary, both autophagy and mitophagy play a crucial role in regulating inflammasome activation by removing inflammatory signals and damaged organelles. This regulation represents a promising therapeutic strategy for targeting the NLRP3 inflammasome in GA. The activation of NLRP3 inflammasome in GA is shown in Fig. 3.

## 5 Natural products targeting the NLRP3 inflammasome to treat GA mechanisms

### 5.1 Targeting the NF-κB/NLRP3 signaling pathway in the priming phase

A variety of natural products have been demonstrated to inhibit the NF-κB signaling pathway at multiple levels. This inhibition regulates the downstream activation of the NLRP3 inflammasome and exerts anti-inflammatory and therapeutic effects against GA. Anethole, an aromatic compound extracted from *Eupatorium*, exhibits anti-inflammatory, antioxidant, and lipid-regulating properties, and is one of the major bioactive constituents in several medicinal plants. In MSU crystal-induced models of acute GA, anethole suppresses NF-κB signaling by simultaneously inhibiting both TLR2 and TLR4 pathways. This action also reduces the activation of the NLRP3 inflammasome, resulting in therapeutic effects [82]. Neoastilbin, primarily isolated from the dried rhizomes of *Smilax glabra*, is a flavonoid compound. It mitigates MSU-induced macrophage inflammation by dual inhibition of the NF-κB pathway and NLRP3 inflammasome activation. This effect is associated with reduced phosphorylation of IκB kinase alpha (IKKα), p65, and IκBα, as well as decreased expression of NLRP3, ASC, and caspase-1 [83]. β-caryophyllene, a bicyclic sesquiterpene compound found in plants like clove and cinnamon, has notable anti-inflammatory and antioxidant properties. Research indicates that it inhibits the TLR4/NF-κB pathway by reducing the expression of TLR4, MyD88, and p65 proteins. This inhibition leads to the suppression of IL-1β secretion, thereby underscoring its role in blocking NLRP3 inflammasome activation [84].

In the MSU crystal-induced model of GA, dioscin, an extract from *Dioscorea spongiosa*, inhibits the TLR4/NF-κB signaling pathway and suppresses NLRP3 inflammasome activation [85]. Similarly, curcumin exerts anti-inflammatory effects by inhibiting TLR4/NF-κB signaling [86,87]. In MSU-stimulated THP-1 macrophages, berberine derived from *Phellodendron chinense* bark downregulates the expression of TLR4, MyD88, IκB kinase beta (IKKβ), and NF-κB, and inhibits caspase-1 activation, thereby reducing the release of inflammatory cytokines [29]. Cichoric acid is a water-soluble phenolic acid derivative from the phenylpropanoid family. Its antioxidant activity arises from a structure that features doubly caffeoyl-modified tartaric acid. Additionally, its inhibitory effect on the NF-κB signaling pathway indicates that it may be a promising candidate for GA-targeted therapy [88]. Pentagalloyl glucose (PGG), a natural polyphenol with XO inhibitory activity, is widely found in medicinal plants such as *Rhus chinensis*, *Paeonia lactiflora*, and *Paeonia suffruticosa*. Research indicates that PGG reduces MSU-induced joint inflammation by targeting TAK1, which in turn blocks NF-κB signaling and prevents the assembly of the NLRP3 inflammasome [36]. Ginsenoside Rb1 alleviates GA by inhibiting the NF-κB/NLRP3 pathway, reducing oxidative stress, and protecting against mitochondrial damage. Its nanoformulation further enhances bioavailability, offering improved therapeutic potential [89].

### 5.2 Targeting the MAPK/NLRP3 signaling pathway in the priming phase

The MAPK signaling pathway plays a crucial role in regulating the expression of inflammatory cytokines and activating the NLRP3 inflammasome in GA. This pathway is a significant target for the anti-inflammatory effects of various natural products. In the MSU crystal-induced model of GA, activation of the JNK signaling pathway occurs alongside NLRP3 inflammasome activation and an increase in inflammatory cytokines, such as IL-1β and TNF-α. Cynarin has been demonstrated to mitigate these pathological changes [90]. Piperine, a natural alkaloid primarily obtained from *Piper nigrum* and *Piper longum,* possesses a range of pharmacological properties, such as anti-inflammatory, anticancer, and immunomodulatory effects. Research shows that piperine reduces MSU-induced joint inflammation by binding to the active site of JNK-1, which suppresses the oligomerization of the NLRP3 inflammasome [91]. Paeonol, a phenolic compound extracted from the root bark of *Paeonia suffruticosa*, is known for its anti-inflammatory properties. It not only inhibits the JNK pathway but also modulates the ERK and p38 signaling pathways. This dual action helps to reduce the inflammatory response induced by MSU crystals [92].

Rutaecarpine, extracted from the dried unripe fruits of *Evodia rutaecarpa*, possesses broad pharmacological activities, with notable anti-inflammatory effects. It alleviates gouty inflammation by directly binding to TNF-α, thereby inhibiting the tumor necrosis factor receptor 1 (TNFR1)/MAPK signaling axis and NLRP3 inflammasome activation [93]. In summary, current research on GA primarily examines the role of the MAPK signaling pathway during the initiation phase of the NLRP3 inflammasome. Nonetheless, the pathway's involvement in PTMs of inflammasome components is still an area that requires further investigation. The mechanisms by which natural products regulate the priming stage of NLRP3 inflammasome are summarized in Table 1 [29,36,[82], [83], [84], [85], [86],[88], [89], [90], [91], [92], [93]].

**Table 1:** Information on natural products targeting the priming phases of the NOD-like receptor family pyrin domain-containing 3 (NLRP3) inflammasome.

Natural product	CAS NO.	Molecular formula	Main sources	*In vivo*/*in vitro*	Modeling methods	Main indicators	Molecular biology mechanisms	Refs.
Berberine	2086-83-1	C_20_H_18_NO_4_^+^	*Phellodendron chinense*	*In vivo*/*In vitro*	*In vitro*: MSU	TLR4↓, p-IKKα/β↓, p-IκBα↓, NF-κB↓, NLRP3↓, caspase-1↓, IL-1β↓, IL-18↓, TNF-α ↓	TLR4/NF-κB pathway inhibition suppressing NLRP3 inflammasome activation	[29]
Pentagalloyl glucose	14937-32-7	C_41_H_32_O_26_	Chinese gallnuts	*In vivo*/*In vitro*	*In vivo*: MSU	p-TAK1↓, p-IκBα↓, p-p65↓, pro-IL-1β↓, NLRP3↓, ASC↓, caspase-1↓, IL-1β↓,	NF-κB pathway inhibition suppressing NLRP3 inflammasome activation	[36]
					*In vitro*: MSU			
Anethole	104-46-1	C_10_H_12_O	*Eupatorium*	*In vivo*	*In vivo*: MSU + polymyxin B	TLR2/4↓, MyD88↓, NF-κB↓, NLRP3↓, caspase-1↓, IL-1β↓, IL-6↓, IL-8↓, TNF-α↓	TLRs/NF-κB pathway inhibition suppressing NLRP3 inflammasome activation	[82]
Neoastilbin	54081-47-9	C_21_H_22_O_11_	*Smilax glabra*	*In vivo*/*In vitro*	*In vivo*: MSU	IKKα↓, p-p65/p65↓, p-IκBα/IκBα↓, NLRP3↓, ASC↓, caspase-1↓, IL-1β↓, IL-6↓, TNF-α↓	NF-κB pathway inhibition suppressing NLRP3 inflammasome activation	[83]
					*In vitro*: LPS + MSU			
β-Caryophyllene	87-44-5	C_15_H_24_	Clove, cinnamon	*In vivo*	*In vivo*: MSU	TLR4↓, MyD88↓, p65↓, pro-IL-1β↓, NLRP3↓, caspase-1↓, ASC↓, IL-1β↓	TLR4/NF-κB pathway inhibition suppressing NLRP3 inflammasome activation	[84]
Dioscin	19057-60-4	C_45_H_72_O_16_	*Dioscorea spongiosa*	*In vivo*/*In vitro*	*In vivo*: MSU	TLR4↓, MyD88↓, p-IKKβ↓, p-p65↓, NLRP3↓, ASC↓, caspase-1↓, IL-1β↓, IL-6↓, TNF-α↓	TLR4/NF-κB pathway inhibition suppressing NLRP3 inflammasome activation	[85]
					*In vitro*: MSU			
Curcumin	458-37-7	C_21_H_20_O_6_	*Curcuma longa*	*In vivo*/*In vitro*	*In vivo:* MSU	TLR4↓, MyD88↓, p-p65↓, p-p50↓, IκBα↑, NLRP3↓, ASC specks↓, caspase-1↓, IL-1β↓, TNF-α↓	TLR4/NF-κB pathway inhibition suppressing NLRP3 inflammasome activation	[86,87]
					*In vitro*: MSU/LPS + MSU			
Cichoric acid	70831-56-0	C_22_H_18_O_12_	*Cichorium intybus*, *Bidens tripartita*, *Echinacea purpurea*	*In vitro*	*In vitro*: MSU	p-p65↓, p-IκBα↓**,** NLRP3↓, ASC↓, caspase-1↓, pro-caspase-1↓, IL-1β↓, TNF-α↓	NF-κB pathway inhibition suppressing NLRP3 inflammasome activation	[88]
Ginsenoside Rb1	41753-43-9	C_54_H_92_O_23_	*Panax ginseng*	*In vivo*	*In vivo*: MSU	IκBα↑, NF-κB↓, NLRP3↓	NF-κB pathway inhibition suppressing NLRP3 inflammasome activation	[89]
Cynarin	30964-13-7	C_25_H_24_O_12_	*Cynara cardunculus*	*In vivo*/*In vitro*	*In vivo*: MSU	p-IKKα/β↓, p-p65↓, p-JNK↓, NLRP3↓, caspase-1↓, IL-1β↓, IL-1β mRNA↓, IL-6 mRNA↓, TNF-α mRNA↓	NF-κB and JNK pathway inhibition suppressing NLRP3 inflammasome activation	[90]
					*In vitro*: LPS + MSU			
Piperine	94-62-2	C_17_H_19_NO_3_	*Piper nigrum*, *Piper longum*	*In vivo*	*In vivo*: MSU	MDA↓, CRP↓, Neutrophil and macrophage exudation↓	MAPK (JNK) and NF-κB (IKKβ) pathway inhibition suppressing NLRP3 inflammasome activation	[91]
Paeonol	552-41-0	C_9_H_10_O_3_	*Paeonia suffruticosa*	*In vitro*	*In vitro*: LPS + MSU	p-JNK↓, p-ERK↓, p-p38↓, p-IKKα/β↓, p-IκBα↓, p-p65↓, NLRP3↓, ASC↓, caspase-1↓, IL-1β↓	MAPK and NF-κB pathway inhibition suppressing NLRP3 inflammasome activation	[92]
Rutaecarpine	84-26-4	C_18_H_13_N_3_O	*Evodia rutaecarpa*	*In vivo*/*In vitro*	*In vivo*: MSU	TNFR1↓, p-p38↓, p-ERK↓, p-JNK↓, p-p65↓, p-IKKα/β↓, NLRP3↓, caspase-1↓, GSDMD-N↓, IL-1β↓, IL-6↓, TNF-α↓	TNFR1/MAPK and NF-κB pathway inhibition suppressing NLRP3 inflammasome activation	[93]
					*In vitro*: LPS + MSU			

ASC: apoptosis-associated speck-like protein containing a caspase recruitment domain; Caspase-1: cysteine-dependent aspartate-directed protease 1; CRP: C-reactive protein; ERK: extracellular signal-regulated kinase; GSDMD-N: N-terminal fragment of gasdermin D; IκBα: inhibitor of nuclear factor kappa-B alpha; IKKα: IκB kinase alpha; IKKβ: IκB kinase beta; IL-1β: interleukin-1 beta; IL-6: interleukin-6; IL-8: interleukin-8; IL-18: interleukin-18; JNK: c-Jun N-terminal kinase; LPS: lipopolysaccharide; MAPK: mitogen-activated protein kinase; MDA: malondialdehyde; MSU: monosodium urate; mRNA: messenger ribonucleic acid; MyD88: myeloid differentiation primary response protein 88; NF-κB: nuclear factor kappa-light-chain-enhancer of activated B cells; NLRP3: NOD-like receptor family pyrin domain containing 3; p50: p50 subunit of NF-κB; p65: p65 subunit of NF-κB; p-ERK: phosphorylated extracellular signal-regulated kinase; p-IKKα: phosphorylated IκB kinase alpha; p-IKKβ: phosphorylated IκB kinase beta; p-IκBα: phosphorylated inhibitor of nuclear factor kappa-B alpha; p-JNK: phosphorylated c-Jun N-terminal kinase; p-p38: phosphorylated p38 mitogen-activated protein kinase; p-p50: phosphorylated p50 subunit of NF-κB; p-p65: phosphorylated p65 subunit of NF-κB; pro-caspase-1: inactive precursor of caspase-1; pro-IL-1β: inactive precursor of interleukin-1beta; p-TAK1: phosphorylated transforming growth factor-beta–activated kinase 1; TLR2: Toll-like receptor 2; TLR4: Toll-like receptor 4; TNF-α: tumor necrosis factor alpha; TNFR1: tumor necrosis factor receptor 1.

### 5.3 Targeting post-translational modifications of NLRP3

In recent years, targeting the post-translational modifications of the NLRP3 inflammasome has emerged as a key mechanism for regulating its activity and inflammatory responses. This approach offers new insights into the treatment of GA. Celastrol is a core active component isolated from traditional Chinese medicine *Tripterygium wilfordii* and other plants of the Celastraceae family. It is a unique five-ring triterpenoid compound with a quinone methyl structure. This compound exhibits multiple biological activities, including significant anti-inflammatory, immunosuppressive, antioxidant, neuroprotective, and metabolic regulatory effects, which have been extensively studied and confirmed. Research indicates that celastrol can inhibit the assembly of the NLRP3 inflammasome and the maturation and release of IL-1β mediated by it. It does this by targeting the deubiquitinating enzyme BRCA1/BRCA2-containing complex subunit 3, effectively blocking the deubiquitination process of the NLRP3 protein at the lysine 63 (K63) site. As a result, celastrol significantly alleviates MSU crystal-induced GA and liver inflammatory damage in mice [94]. This study elucidates the mechanism by which celastrol exerts its anti-inflammatory effects by regulating the ubiquitination modification of NLRP3. This finding offers new intervention targets and strategies for the treatment of GA.

### 5.4 Targeting ROS generation and scavenging

Oxidative stress plays a crucial role in the development of GA, and various natural compounds reduce ROS production and inhibit inflammasome activation by activating the Nrf2 pathway, regulating mitochondrial function, and modulating the TXNIP/NLRP3 axis. Eucalyptol, an active compound extracted from *Eucalyptus* leaves, activates the Nrf2/heme oxygenase-1 (HO-1) pathway. This activation restores the body's endogenous antioxidant capacity and increases the activity of superoxide dismutase and glutathione peroxidase in joint tissues. As a result, eucalyptol suppresses NLRP3 inflammasome activation and the release of inflammatory cytokines [95]. Similarly, andrographolide from *Andrographis paniculata* and palmatine from *Corydalis yanhusuo* inhibit MSU crystal-induced ROS production by upregulating HO-1 expression [96,97]. Under normal conditions, Kelch-like ECH-associated protein 1 (Keap1) acts as a negative regulator of Nrf2, ensuring its basal stability. Phillyrin, an active component derived from the dried fruits of *Forsythia suspensa*, can disrupt the interaction between Keap1 and Nrf2. This disruption leads to a reduction in ROS levels and neutrophil extracellular trap formation, suppresses NLRP3 inflammasome activation, and alleviates joint swelling and neutrophil infiltration in mouse models [98]. Although some studies have only demonstrated that increasing antioxidant enzyme activity to alleviate ROS can inhibit NLRP3 inflammasome activation, this still underscores the therapeutic potential of targeting the ROS pathway in GA. Numerous natural products have been identified with such effects, including rutin (widely present in plants and fruits), tetrahydropalmatine from *Corydalis yanhusuo*, and kaempferol [52,99,100].

In GA, mtROS are a significant source of intracellular ROS, and their production is closely linked to mitochondrial dysfunction. Consequently, modulating mitochondrial function and maintaining its stability are crucial for therapeutic strategies. In MSU-stimulated THP-1 macrophages, lipoxin A4, a natural endogenous compound, enhances mitochondrial function and reduces mtROS generation by targeting the Nrf2/Krueppel-like factor 9 (Klf9)/thioredoxin reductase 2 (TXNRD2) pathway. This action effectively blocks the assembly and activation of the NLRP3 inflammasome, leading to a significant reduction in MSU crystal-induced joint inflammation [101]. Obovatol, an active constituent derived from *Magnolia obovata* leaves, has been extensively studied for its antibacterial, anti-inflammatory, and neuroprotective properties. Research indicates that obovatol mitigates gouty inflammation by suppressing mtROS generation, which in turn inhibits NLRP3 inflammasome activation [102]. Baeckein E is a flavonoid compound that is present in the myrtle plant *Baeckea frutescens* [17]. In addition to blocking the binding between ASC and pro-caspase-1, baeckein E can also ameliorate mitochondrial damage (restoring mitochondrial membrane potential and mitochondrial number) to reduce mtROS, thereby improving MSU crystal induced GA [17]. Moreover, diallyl trisulfide, a compound derived from *Allium sativum*, inhibits NOX3/4-dependent mtROS generation in MSU treated macrophages. It also blocks NLRP3 inflammasome assembly and the maturation and release of IL-1β. This provides novel mechanistic insights into the use of natural products that target the NLRP3 inflammasome for GA therapy [48].

Under ROS stimulation, TXNIP dissociates from TRX and binds to NLRP3, facilitating inflammasome assembly and IL-1β secretion, suggesting that TXNIP may serve as a key regulatory node in NLRP3 inflammasome activation. Catechin, a phenolic compound found in tea and other plants, significantly reduces MSU-induced mtROS levels. It also downregulates the expression of the TXNIP/NLRP3 complex, which inhibits the activation of the NLRP3 inflammasome [103]. Furthermore, evodiamine, a quinazoline alkaloid extracted from *Tetradium ruticarpum*, and berberine, an isoquinoline alkaloid derived from traditional Chinese medicines such as *Coptis chinensis* and *Phellodendron chinense*, inhibit the interaction between TXNIP and NLRP3 by modulating the ROS/TXNIP/NLRP3 axis and activating the Nrf2 antioxidant pathway, respectively. These actions effectively block NLRP3 inflammasome activation and alleviate GA symptoms [104,105].

### 5.5 Targeting mtDNA release

An increasing number of studies highlight the potential of natural products to prevent the release of mtDNA into the cytoplasm. This prevention inhibits the activation of the NLRP3 inflammasome and mitigates inflammation associated with GA. Although research in this area is still limited, accumulating evidence suggests that targeting mtDNA could offer promising therapeutic potential for GA. Green tea, recognized as the earliest type of tea in Chinese history, has a long-established tradition of production and use. A major polyphenolic component of *Camellia sinensis*, epigallocatechin-3-gallate (EGCG) has attracted considerable research interest due to its impressive anti-inflammatory properties. Studies have shown that EGCG effectively reduces inflammation in a murine model of acute GA induced by MSU crystals. This effect occurs through mechanisms that inhibit mtDNA de novo synthesis and ROS production, ultimately leading to a reduction in NLRP3 inflammasome activation caused by oxidized mtDNA [106].

### 5.6 Targeting lysosomal homeostasis

Lysosomal stability is crucial for the activation of the NLRP3 inflammasome. Natural products can exert anti-inflammatory effects by either protecting the lysosomal structure or suppressing the expression of cathepsin B. Procyanidin B2 (PCB2), a polyphenolic compound extracted from grape seeds, has been shown to have anti-inflammatory effects in multiple studies. Evidence suggests that PCB2 significantly inhibits the activation of the NLRP3 inflammasome induced by MSU crystals, as well as the subsequent secretion of downstream cytokines, primarily by downregulating the expression of cathepsin B [107]. Moreover, Euphorbia factor L2, a natural compound derived from the seeds of *Euphorbia* species within the Euphorbiaceae family, has been shown to specifically repair MSU crystal-induced lysosomal membrane damage. This protective effect effectively blocks NLRP3 inflammasome activation, thereby markedly reducing GA-associated inflammatory responses and joint swelling [108].

### 5.7 Targeting ion homeostasis

In recent years, a growing body of research has demonstrated that various natural products can effectively inhibit the activation of the NLRP3 inflammasome induced by MSU crystals by disrupting ion homeostasis, thereby alleviating GA. One such product, benzoylmesaconine, is an alkaloid extracted from the traditional Chinese medicinal herb *Aconitum carmichaelii*. It exhibits a wide range of biological activities, particularly in analgesia. Studies indicate that benzoylmesaconine inhibits NLRP3 inflammasome activation by suppressing the efflux of intracellular potassium ions, disrupting ASC oligomerization, and interfering with the interaction between NLRP3 and NEK7. These actions reduce caspase-1 activation, IL-1β maturation and release, and GSDMD cleavage, ultimately leading to the alleviation of joint swelling and pain in murine models of GA [109]. Kynurenic acid (KA) is an endogenous tryptophan metabolite with immunomodulatory, antioxidant and anti-inflammatory properties. Research has shown that KA selectively inhibits MSU crystal-induced Ca^2+^ influx by activating the G protein-coupled receptor 35 (GPR35). This activation prevents mitochondrial dysfunction and the production of ROS, ultimately blocking the assembly and activation of the NLRP3 inflammasome [71].

### 5.8 Targeting autophagy and mitophagy

Natural products that target the autophagy pathway to inhibit NLRP3 inflammasome activation have shown therapeutic potential in GA. This potential mainly arises from their capacity to eliminate the activating signals associated with the NLRP3 inflammasome. Nobiletin, a flavonoid compound widely found in the peels of citrus plants such as *Citrus reticulata* and *Citrus unshiu*, exhibits multiple biological activities, including anticoagulant, antithrombotic, anticancer, and anti-allergic effects. Studies have shown that, in the context of inflammation, nobiletin promotes autophagy by activating the AMPK/mTOR pathway. This activation suppresses NLRP3 inflammasome activation and significantly alleviates inflammatory responses in MSU-induced GA in both mice and THP-1 macrophages [77]. Leojaponin, a bioactive diterpene compound, is primarily isolated from the ethanol extract of the herb *Leonurus japonicus*. Research indicates that leojaponin enhances autophagy function by upregulating Raptor phosphorylation. This action effectively inhibits the assembly of the NLRP3 inflammasome and the maturation of downstream IL-1β. Consequently, there is a significant reduction in acute inflammatory responses in MSU-induced GA in mice [110].

Resveratrol, a natural stilbene compound mainly sourced from *Reynouria japonica*, is also found in various fruits and nuts. Research has shown that resveratrol enhances mitophagy by activating the Pink1/Parkin pathway, which helps clear damaged mitochondria. Additionally, it suppresses NLRP3 inflammasome activation, leading to a reduction in joint swelling and pathological damage in GA animal models [111].

### 5.9 Targeting the effector phase of NLRP3 inflammasome activation

Targeting the effector phase of NLRP3 inflammasome activation is a crucial strategy for interrupting downstream inflammatory signaling and alleviating GA symptoms. For example, coptisine not only inhibits the interaction between ASC and pro-caspase-1, preventing NLRP3 inflammasome assembly, but also directly suppresses caspase-1 activity. This dual action reduces IL-1β secretion and mitigates inflammation associated with GA [112]. Similarly, sennoside A exhibits comparable inhibitory effects [113]. Moreover, benzoylmesaconine has been shown to not affect GSDMD expression, but instead inhibits its cleavage into the GSDMD-N fragment, thereby reducing inflammatory cytokine release [109]. However, this effect may stem from the suppression of upstream NLRP3 inflammasome signals or caspase-1 activity, rather than from a direct interaction with GSDMD. The regulatory mechanisms of natural products targeting the activation stage of the NLRP3 inflammasome are summarized in Table 2 [17,48,52,71,77,[94], [95], [96], [97], [98], [99], [100], [101], [102], [103], [104], [105], [106], [107], [108], [109], [110], [111], [112], [113]].

**Table 2:** Information on natural products targeting the activation phases of the NOD-like receptor family pyrin domain-containing 3 (NLRP3) inflammasome.

Natural product	CAS NO.	Molecular formula	Main sources	*In vivo*/*in vitro*	Modeling methods	Main indicators	Molecular biology mechanisms	Refs.
Baeckein E	–	C_16_H_14_O_5_	*Baeckea frutescens*	*In vivo*/*in vitro*	*In vivo*: MSU	MMP↑, Mitochondrial↑, ROS↓, ASC oligomerization↓, NLRP3↓, GSDMD-N↓, caspase-1↓, IL-1β↓	Inhibition of mitochondrial oxidative damage/ROS suppressing NLRP3 activation	[17]
					*In vitro*: LPS + ATP			
Diallyl trisulfide	2050-87-5	C_6_H_10_S_3_	*Allium sativum*	*In vivo*/*in vitro*	*In vivo*: MSU	MMP↑, mtROS↓, NOX3/4↓, ASC oligomerization↓, IL-1β↓, caspase-1↓	NOX3/4 inhibition suppressing mtROS and NLRP3 activation	[48]
					*In vitro*: LPS + ATP			
Rutin	153-18-4	C_27_H_30_O_16_	*Sophora japonica*	*In vivo*/*in vitro*	*In vivo*: High-purine diet + 10 % fructose drinking water	XO↓, UA↓, MDA↓, SOD↑, GSH↑, ROS↓, NLRP3↓, ASC↓, caspase-1↓, IL-1β↓, IL-18↓, TNF-α↓, IL-8↓	Reducing XO activity, inhibiting ROS production and NLRP3 inflammasome activation	[52]
					*In vitro*: UA			
Kynurenic acid	492-27-3	C_10_H_7_NO_3_	Trptophan metabolites	*In vivo*/*in vitro*	*In vivo*: MSU	Intracellular Ca^2+^↓, mtROS↓, caspase-1↓, IL-1β↓	GPR35 activation decreasing Ca^2+^ mobilization, thereby suppressing NLRP3 activation	[71]
					*In vitro*: LPS/LPS + MSU			
Nobiletin	478-01-3	C_21_H_22_O_8_	Citrus fruits	*In vivo*/*in vitro*	*In vivo*: MSU	p-AMPK↑, p-mTOR↓, LC3-II/I↑, Beclin-1↑, p62↓, NLRP3↓, ASC↓, caspase-1↓, IL-1β↓, IL-18↓, IL-6↓, TNF-α↓,	AMPK/mTOR-mediated autophagy activation suppressing NLRP3 inflammasome activation	[77]
					*In vitro*: LPS + MSU			
Celastrol	34157-83-0	C_29_H_38_O_4_	*Tripterygium wilfordii, Celastrus orbiculatus*	*In vitro*	*In vitro*: MSU	K63 deubiquitination↓, caspase-1↓, IL-1β↓	Inhibition of K63-linked deubiquitination of NLRP3, thereby suppressing inflammasome activation	[94]
Eucalyptol	470-82-6	C_10_H_18_O	*Eucalyptus* essential oils	*In vivo*/*in vitro*	*In vivo*: MSU	Nrf2↑, HO-1↑, SOD↑, GSH-Px↑, MDA↓, ROS↓, NLRP3↓, caspase-1↓, IL-1β↓, TNF-α↓, IL-6↓, CXCL2↓	Nrf2/HO-1 activation and ROS reduction suppressing NLRP3 inflammasome activation	[95]
					*In vitro*: MSU			
Andrographolide	5508-58-7	C_20_H_30_O_5_	*Andrographis paniculata*	*In vivo*/*in vitro*	*In vivo*: MSU	HO-1↑, ROS↓, NLRP3-ASC↓, NLRP3↓, pro-IL-1β↓, caspase-1↓, IL-1β↓	Nrf2/HO-1 activation and ROS reduction suppressing NLRP3 inflammasome activation	[96]
					*In vitro*: LPS + MSU			
Palmatine	3486-67-7	C_21_H_22_NO_4_^+^	*Corydalis yanhusuo*	*In vivo*/*in vitro*	*In vivo*: MSU	SOD↑, GSH↑, MDA↓, NLRP3↓, ASC↓, caspase-1↓, IL-1β↓, TNF-α↓, IL-6↓, IL-18↓	Nrf2/HO-1 activation suppressing NLRP3 inflammasome activation	[97]
					*In vitro*: LPS + MSU			
Phillyrin	487-41-2	C_27_H_34_O_11_	*Forsythia suspensa*	*In vivo*/*in vitro*	*In vivo*: MSU	Nrf2↑, HO-1↑, MDA↓, SOD↑, GSH↑, ROS↓, NLRP3↓, ASC↓, caspase-1↓, IL-1β↓, TNF-α↓, IL-6↓	Nrf2/HO-1 activation suppressing NLRP3 inflammasome activation	[98]
					*In vitro*: MSU			
Tetrahydropalmatine	483-14-7	C_21_H_25_NO_4_	*Corydalis yanhusuo*	*In vivo*/*in vitro*	*In vivo*: MSU	SOD↑, GSH-Px↑, MDA↓, ROS↓, NLRP3↓, ASC↓, caspase-1↓, IL-1β↓, IL-18↓, IL-6↓, TNF-α↓	ROS reduction suppressing NLRP3 inflammasome activation	[99]
					*In vitro*: LPS + MSU			
Kaempferol	520-18-3	C_15_H_10_O_6_	Widely present in fruits, vegetables, Chinese herbal medicines and other natural plants	*In vivo*/*in vitro*	*In vivo*: xanthine + MSU	XO↓, UA↓, SOD↑, GSH-Px↑, MDA↓, ROS↓, NLRP3↓, caspase-1↓, ASC↓, IL-1β↓, IL-6↓, TNF-α↓	Inhibition of XO activity, reduction of ROS production, and suppression of NLRP3 inflammasome activation	[100]
					*In vitro*: LPS + MSU/MSU			
Lipoxin A4	89663-86-5	C_20_H_32_O_5_	Human arachidonic acid metabolites	*In vivo*/*in vitro*	*In vivo*: MSU	Nrf2↓, Klf9↓, TXNRD2↑, ROS↓, NADPH oxidase activity↓, ASC speck↓, ASC oligomerization↓, ASC-NLRP3↓, caspase-1↓, IL-1β↓, GSDMD-N↓	Inhibition of the Nrf2/Klf9/TXNRD2 signaling pathway to reduce ROS accumulation, thereby suppressing NLRP3 inflammasome activation	[101]
					*In vitro*: LPS + MSU			
Obovatol	83864-78-2	C_18_H_18_O_3_	*Magnolia obovata*	*In vivo*/*in vitro*	*In vivo*: MSU	mtROS↓, NLRP3↓, caspase-1↓, IL-1β↓, IL-18↓	Inhibiting mitochondrial ROS generation to suppress NLRP3 inflammasome activation	[102]
					*In vitro*: LPS			
Catechin	154-23-4	C_15_H_14_O_6_	Tea types	*In vivo*/*in vitro*	*In vivo*: MSU	mtROS↓, MMP↑, intracellular Ca^2+^↓, NLRP3-TXNIP↓, IL-1β↓, IL-6↓	Inhibiting mtROS accumulation, suppressing Ca^2+^ influx, and blocking TXNIP-mediated NLRP3 inflammasome activation	[103]
					*In vitro*: MSU			
Evodiamine	518-17-2	C_19_H_17_N_3_O	*Tetradium ruticarpum*	*In vivo*	*In vivo*: MSU	XO↓, MDA↓, SOD↑, ROS↓, TXNIP↓, NLRP3↓, ASC↓, pro-caspase-1↓, caspase-1↓, IL-1β↓, IL-18↓, TNF-α↓	Inhibition of ROS production and blockade of the ROS/TXNIP/NLRP3 pathway to suppress NLRP3 inflammasome activation	[104]
Berberine	2086-83-1	C_20_H_18_NO_4_^+^	*Coptis chinensis, Phellodendri chinensis*	*In vivo*/*in vitro*	*In vivo*: MSU	TLR4↓, IKKα/β↑, p-IKKα/β↓, p-IκBα↓, NF-κB (p50/65)↓, NLRP3↓, IL-1β↓, IL-18↓, Keap1↓, Nrf2↑, HO-1↑, SOD↑, ROS↓, TXNIP↓, NLRP3↓, caspase-1↓, IL-1β↓, TNF-α↓	Activation of the Nrf2 pathway to enhance antioxidant capacity, and inhibition of TXNIP-mediated NLRP3 inflammasome activation	[105]
					*In vitro*: MSU			
Epigallocatechin-3-gallate	989-51-5	C_22_H_18_O_11_	*Camellia sinensis*	*In vivo*	*In vivo*: MSU	ROS↓, mtDNA↓, caspase-1↓, IL-1β↓	Inhibition of mtDNA synthesis and ROS generation to suppress NLRP3 inflammasome activation	[106]
Procyanidin B2	29106-49-8	C_30_H_26_O_12_	Grape seeds	*In vivo*/*in vitro*	*In vivo*: MSU	Cathepsin B↓, NLRP3↓, IL-1β↓	Reduction of Cathepsin B release and inhibition of NLRP3 inflammasome activation	[107]
					*In vitro*: LPS + MSU			
Euphorbia Factor L2	218916-51-9	C_38_H_42_O_9_	*Euphorbia lathyris*	*In vivo*/*in vitro*	*In vivo*: MSU	Lysosomal Membrane Integrity↑, NLRP3↓, ASC-NLRP3↓, caspase-1↓, IL-1β↓, IL-6↓	Inhibition of lysosomal damage to suppress NLRP3 inflammasome activation	[108]
					*In vitro*: LPS + MSU			
Benzoylmesaconine	63238-67-5	C_31_H_43_NO_10_	*Aconitum carmichaelii*	*In vivo*/*in vitro*	*In vivo*: MSU	Intracellular K^+^ efflux↓, ASC speck↓, NLRP3-NEK7↓, IL-1β↓, TNF-α↓, GSDMD cleavage↓	Inhibition of intracellular K^+^ efflux and NLRP3 inflammasome assembly to suppress inflammasome activation	[109]
					*In vitro*: LPS + MSU			
Leojaponin	864817-63-0	C_20_H_26_O_3_	*Leonurus japonicus*	*In vivo*/*in vitro*	*In vivo*: MSU	p-Raptor↑, p-mTOR↓, p62↓, LC3B-II↑, ASC specks↓, caspase-1↓, GSDMD cleavage↓, LDH↓, IL-1β↓	Upregulation of raptor phosphorylation to restore autophagy and suppress NLRP3 inflammasome activation	[110]
					*In vitro*: LPS + MSU			
Resveratrol	501-36-0	C_14_H_12_O_3_	*Reynouria japonica*	*In vivo*/*in vitro*	*In vivo*: MSU	MMP↑, LC3B-II↑, Parkin↑, TOMM20↓, p62↓, caspase-1↓, IL-1β↓, IL-18↓	Mitophagy activation suppressing NLRP3 activation	[111]
					*In vitro*: LPS + MSU			
Coptisine	3486-66-6	C_19_H_14_NO_4_^+^	*Coptis chinensis*	*In vivo*/*in vitro*	*In vivo*: MSU	NLRP3↓, ASC-pro-caspase-1↓, caspase-1 activity↓, IL-1β↓, IL-18↓	Inhibition of caspase-1 suppressing NLRP3 inflammasome activation	[112]
					*In vitro*: LPS + ATP/MSU/Nigericin			
Sennoside A	81-27-6	C_42_H_38_O_20_	*Rheum palmatum*	*In vivo*/*in vitro*	*In vivo*: MSU	NLRP3↓, ASC-pro-caspase-1↓, caspase-1 activity↓, IL-1β↓, IL-18↓	Inhibition of caspase-1 suppressing NLRP3 inflammasome activation	[113]
					*In vitro*: LPS + ATP/MSU/Nigericin			

AMPK: AMP-activated protein kinase; ASC: apoptosis-associated speck-like protein containing a caspase recruitment domain; ATP: adenosine triphosphate; Beclin-1: beclin-1 autophagy-related protein; Cathepsin B: lysosomal cysteine protease B; caspase-1: cysteine-dependent aspartate-directed protease 1; CXCL2: C-X-C motif chemokine ligand 2; GPR35: G protein-coupled receptor 35; GSDMD: gasdermin D; GSDMD-N: N-terminal fragment of gasdermin D; GSH: glutathione; GSH-Px: glutathione peroxidase; HO-1: heme oxygenase 1; IκBα: inhibitor of nuclear factor kappa-B alpha; IKKα/β: IκB kinase alpha/beta; IL-1β: interleukin-1 beta; IL-6: interleukin-6; IL-8: interleukin-8; IL-18: interleukin-18; Keap1: Kelch-like ECH-associated protein 1; Klf9: Kruppel-like factor 9; K63: lysine 63; LC3B-II: microtubule-associated proteins 1A/1B light chain 3B type II; LC3-II/I: microtubule-associated protein 1 light chain 3 type II/I; LDH: lactate dehydrogenase; LPS: lipopolysaccharide; MDA: malondialdehyde; MMP: mitochondrial membrane potential; mRNA: messenger RNA; MSU: monosodium urate; mtDNA: mitochondrial DNA; mtROS: mitochondrial reactive oxygen species; mTOR: mechanistic target of rapamycin; NADPH: nicotinamide adenine dinucleotide phosphate; NF-κB: nuclear factor kappa-light-chain-enhancer of activated B cells; NEK7: NIMA-related kinase 7; NLRP3: NOD-like receptor family pyrin domain containing 3; NOX3/4: NADPH oxidase isoforms 3 and 4; Nrf2: nuclear factor erythroid 2–related factor 2; p-AMPK: phosphorylated AMPK; p50: p50 subunit of NF-κB; p62: sequestosome-1; p65: p65 subunit of NF-κB; p-IKKα/β: phosphorylated IκB kinase alpha/beta; p-IκBα: phosphorylated inhibitor of nuclear factor kappa-B alpha; p-mTOR: phosphorylated mechanistic target of rapamycin; p-Raptor: phosphorylated regulatory-associated protein of mTOR; pro-caspase-1: inactive precursor of caspase-1; pro-IL-1β: inactive precursor of interleukin-1 beta; ROS: reactive oxygen species; SOD: superoxide dismutase; TLR4: Toll-like receptor 4; TNF-α: tumor necrosis factor alpha; TOMM20: translocase of outer mitochondrial membrane 20; TXNIP: thioredoxin-interacting protein; TXNRD2: thioredoxin reductase 2; UA: uric acid; XO: xanthine oxidase.

### 5.10 Direct targeting of the NLRP3 inflammasome

**Fig. 4:**
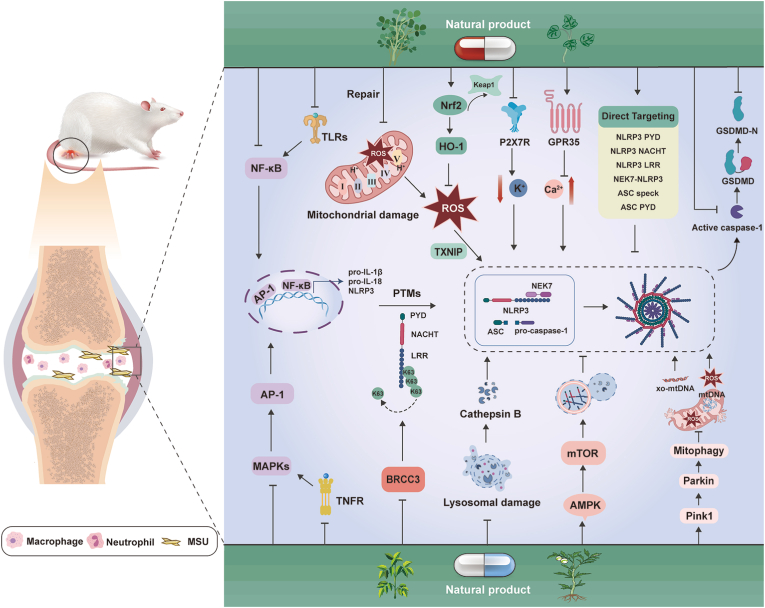
Mechanism of action of natural products. AMPK: AMP-activated protein kinase; AP-1: activator protein 1; ASC: apoptosis-associated speck-like protein containing a caspase recruitment domain; BRCC3: BRCA1/BRCA2-containing complex subunit 3; GPR35: G protein-coupled receptor 35; GSDMD: gasdermin D; GSDMD-N: N-terminal fragment of gasdermin D; HO-1: heme oxygenase-1; K63: Lys63-linked ubiquitinatio; Keap1: Kelch-like ECH-associated protein 1; LRR: leucine-rich repeat; MAPKs: mitogen-activated protein kinases; MSU: monosodium urate; mtDNA: mitochondrial DNA; mTOR: mechanistic target of rapamycin; NACHT: nucleotide-binding and oligomerization domain shared by NAIP, CIITA, HET-E, and TP1 proteins; NEK7: NIMA-related kinase 7; NF-κB: nuclear factor kappa-light-chain-enhancer of activated B cells; NLRP3: NOD-like receptor family pyrin domain containing 3; Nrf2: nuclear factor erythroid 2–related factor 2; P2X7R: P2X7 receptor; Pink1: PTEN-induced kinase 1; pro-IL-1β: pro-interleukin-1 beta; pro-IL-18: pro-interleukin-18; PTMs: post-translational modifications; PYD: pyrin domain; ROS: reactive oxygen species; TLRs: Toll-like receptors; TNFR: tumor necrosis factor receptor; TXNIP: thioredoxin-interacting protein; xo-mtDNA: oxidized mitochondrial DNA.

Directly targeting critical functional domains of the NLRP3 inflammasome or its protein-protein interactions to block downstream inflammatory cascades aligns with the principles of targeted therapy in precision medicine. Natural products that target various domains or protein interactions demonstrate multi-level inhibition of NLRP3 inflammasome at the molecular level. These mechanisms include the inhibition of NLRP3's ATPase activity, targeting its PYD or LRR domains, blocking the NLRP3-NEK7 interaction, and interfering with ASC complex formation. Together, these mechanisms form the structural basis for the precise regulation of NLRP3 inflammasome, offering a valuable source of natural lead compounds for the treatment of GA. Accordingly, this review summarizes 18 representative natural products [[112], [113], [114], [115], [116], [117], [118], [119], [120], [121], [122], [123], [124], [125], [126], [127], [128], [129], [130]] and summarized their specific targets and molecular mechanisms in Table 3 [[112], [113], [114], [115], [116], [117], [118], [119], [120], [121], [122], [123], [124], [125], [126], [127], [128], [129], [130]]. The mechanisms of natural products are illustrated in Fig. 4.

**Table 3:** Information on natural products directly targeting NOD-like receptor family pyrin domain containing 3 (NLRP3) inflammasome components.

Natural products	CAS NO.	Molecular formula	Main sources	*In vivo*/*in vitro*	Modeling methods	Molecular biology mechanisms	Refs.
Coptisine	3486-66-6	C_19_H_14_NO_4_^+^	*Coptis chinensis*	*In vivo*/*in vitro*	*In vivo*: MSU	Inhibition of the interaction between ASC and pro-caspase-1 to block inflammasome assembly	[112]
					*In vitro*: LPS + nigericin/ATP/MSU		
Sennoside A	81-27-6	C_42_H_38_O_20_	*Rheum palmatum*	*In vivo*/*in vitro*	*In vivo:* MSU	Inhibition of the interaction between ASC and pro-caspase-1 to block inflammasome assembly	[113]
					*In vitro*: LPS + nigericin/ATP/MSU		
Erianin	95041-90-0	C_20_H_22_O_5_	*Dendrobium chrysotoxum*	*In vivo*/*in vitro*	*In vivo*: MSU	Inhibition of NLRP3 ATPase activity by targeting the Walker A motif within its NACHT domain	[114]
					*In vitro*: LPS + MSU		
β-Carotene	7235-40-7	C_40_H_56_	Fruits, vegetables, etc.	*In vivo*/*in vitro*	*In vivo*: MSU	Direct binding to the PYD domain of NLRP3 to block its interaction with ASC	[115]
					*In vitro*: MSU/ATP/nigericin		
Costunolide	553-21-9	C_15_H_20_O_2_	*Saussurea lappa*	*In vivo*/*in vitro*	*In vivo*: MSU	Covalent targeting of the Cys598 residue within the NACHT domain to inhibit NLRP3 ATPase activity	[116]
					*In vitro*: LPS + ATP/nigericin/alum		
Octyl gallate	1034-01-1	C_15_H_22_O_5_	Chinese gall	*In vivo*/*in vitro*	*In vivo*: MSU	Direct binding to the LRR domain of NLRP3 to block its oligomerization	[117]
					*In vitro*: LPS + nigericin/ATP/MSU/IMQ		
Anemoside B4	129741-57-7	C_59_H_96_O_26_	*Pulsatilla chinensis*	*In vivo*/*in vitro*	*In vivo*: MSU	Inhibition of NEK7–NLRP3 interaction via directly binding to NEK7 Ser46 and Phe168 residues	[118]
					*In vitro:* MSU		
Oridonin	28957-04-2	C_20_H_28_O_6_	*Rabdosia rubescens*	*In vivo*/*in vitro*	*In vivo:* MSU	Inhibition of NLRP3–NEK7 interaction via covalent binding to the Cys279 residue in the NACHT domain	[119]
					*In vitro*: LPS + nigericin/ATP/MSU		
Britannin	33627-28-0	C_19_H_26_O_7_	*Inula japonica*	*In vivo*/*in vitro*	*In vivo:* MSU	Inhibition of NLRP3–NEK7 interaction via binding to the Arg335/Gly271 sites in the NACHT domain	[120]
					*In vitro*: LPS + ATP		
Pristimerin	1258-84-0	C_30_H_40_O_4_	*Celastraceae*, *Hippocrateaceae*	*In vivo*/*in vitro*	*In vivo*: MSU	Inhibition of NLRP3–NEK7 interaction via covalent bonding between the α,β-unsaturated carbonyl group and cysteine residues in the NACHT domain	[121]
					*In vitro*: LPS + nigericin/ATP/MSU		
Artemisinin	63968-64-9	C_15_H_22_O_5_	*Artemisia annua*	*In vivo*/*in vitro*	*In vivo*: MSU	Inhibition of the NEK7–NLRP3 interaction	[122]
					*In vitro*: LPS + MSU		
Hypocrellin A	77029-83-5	C_30_H_26_O_10_	*Hypocrella bambusae*	*In vivo*/*in vitro*	*In vitro*: LPS + nigericin/ATP/MSU	Inhibition of NLRP3–NEK7 interaction via direct binding to the Lys232, Glu260, Asp305, and Arg351 residues in the NACHT domain	[123]
Alantolactone	546-43-0	C_15_H_20_O_2_	*Saussurea lappa*	*In vivo*/*in vitro*	*In vivo*: MSU	Inhibition of NLRP3-NEK7 interaction via direct binding to the Arg335 residue in the NACHT domain of NLRP3	[124]
					*In vitro*: LPS + ATP/alum/MSU		
Licochalcone B	58749-23-8	C_16_H_14_O_5_	*Glycyrrhiza uralensis*	*In vivo*/*in vitro*	*In vivo*: MSU	Inhibition of NLRP3–NEK7 interaction via specific binding to the NEK7 protein	[125]
					*In vitro:* LPS + nigericin/ATP/MSU		
Chloranthalactone B	66395-03-7	C_15_H_16_O_3_	*Sarcandra glabra*	*In vivo*/*in vitro*	*In vivo*: MSU	Inhibition of NLRP3–NEK7 interaction via direct binding to the Cys279 residue in the NACHT domain of NLRP3	[126]
					*In vitro*: LPS + nigericin/ATP/MSU		
Theaflavin	4670-05-7	C_29_H_24_O_12_	*Camellia sinensis*	*In vivo*/*in vitro*	*In vivo*: MSU	Inhibition of the NEK7–NLRP3 interaction	[127]
					*In vitro*: LPS + nigericin/ATP/MSU		
Rhein	478-43-3	C_15_H_8_O_6_	*Rheum palmatum*	*In vitro*	*In vitro:* MSU	Inhibition of ASC speck formation and caspase-1 activity	[128]
Caffeic acid phenethyl ester	104594-70-9	C_17_H_16_O_4_	Honeybee propolis	*In vivo*/*in vitro*	*In vivo*: MSU	Inhibition of ASC speck formation via direct binding to ASC	[129]
					*In vitro*: LPS + nigericin/ATP/MSU		
Gallic acid	149-91-7	C_7_H_6_O_5_	Tea types, grapes, pomegranates and peonies	*In vivo*/*in vitro*	*In vivo*: MSU	Inhibition of the NEK7–NLRP3 interaction	[130]
					*In vitro*: LPS + nigericin/ATP/MSU		

ASC: apoptosis-associated speck-like protein containing a caspase recruitment domain; ATP: adenosine triphosphate; IMQ: imiquimod; LPS: lipopolysaccharide; LRR: leucine-rich repeat; MSU: monosodium urate; NACHT: nucleotide-binding and oligomerization domain shared by NAIP, CIITA, HET-E, and TP1 proteins; NEK7: NIMA-related kinase 7; NLRP3: NOD-like receptor family pyrin domain containing 3; PYD: pyrin domain.

#### 5.10.1 Targeting the ATPase activity of NLRP3

The NACHT domain of the NLRP3 protein features Walker A and Walker B motifs, which are essential for ATP binding and hydrolysis. The process of NLRP3 oligomerization relies on conformational changes in the NACHT domain that occur upon ATP binding, exposing the oligomerization interface and promoting inflammasome assembly. Consequently, targeting the NACHT domain to inhibit its ATPase activity offers a direct strategy to prevent NLRP3 inflammasome activation.

Several natural products have been shown to suppress NLRP3 inflammasome activation through this mechanism. Erianin inhibits ATP hydrolysis and oligomerization of NLRP3 by targeting the Walker A motif within its NACHT domain [114]. The sesquiterpene lactone costunolide covalently binds to the Cys598 residue in the NACHT domain of NLRP3 through its α-methylene-γ-butyrolactone motif. This interaction inhibits ATPase activity and stabilizes the autoinhibited conformation of NLRP3, effectively preventing oligomerization and downstream inflammasome assembly [116]. These findings indicate that the NACHT domain functions as both the catalytic core for ATP binding and hydrolysis and a critical target for the precise small-molecule modulation of NLRP3 activity.

#### 5.10.2 Targeting the PYD or LRR domains of NLRP3

The NLRP3 protein features a PYD domain at its N-terminus and an LRR domain at its C-terminus. The PYD domain facilitates the interaction between NLRP3 and ASC, while the LRR domain helps maintain NLRP3's autoinhibited conformation and plays a role in sensing upstream signals. Notably, β-carotene can directly target the PYD domain of NLRP3, specifically inhibiting its interaction with ASC [115]. Molecular modeling has revealed that a hydrophobic groove composed of five amino acid residues, Ala^69^, Val^72^, Trp^73^, Tyr^84^, and Glu^91^, serves as the key binding motif through which β-carotene interacts with the PYD domain of NLRP3 to exert its inhibitory function. In addition, octyl gallate can directly bind to the LRR domain of NLRP3, irreversibly suppressing inflammasome assembly [117]. Natural products can stabilize the autoinhibited state of NLRP3 or block ASC recruitment by binding to these domains. This action inhibits inflammasome activation during the early stages of assembly.

#### 5.10.3 Targeting the NLRP3-NEK7 interaction

The full activation of NLRP3 relies on its interaction with the mitotic kinase NEK7. NEK7 recognizes specific residues within the NACHT domain of NLRP3, stabilizing its oligomeric conformation and functioning as a crucial cofactor for inflammasome assembly. By blocking this protein–protein interaction (PPI) selective inhibition of NLRP3 inflammasome activation can be achieved without affecting other members of the NLR family. Numerous studies have demonstrated that natural products can directly target either NEK7 or NLRP3, precisely modulating their interaction to suppress the assembly and activation of the NLRP3 inflammasome.

Certain compounds act directly on the NACHT domain of NLRP3, forming covalent bonds that prevent NEK7 binding. For example, Oridonin covalently binds to the Cys279 residue of NLRP3, thereby inhibiting the interaction between NEK7 and NLRP3 [119]. Britannin directly interacts with Arg335 and Gly271 of the NLRP3 NACHT domain [120]. Pristimerin, through its α,β-unsaturated carbonyl group, forms a covalent bond with cysteine residues within the NACHT domain [121]. Hypocrellin A binds to key residues Lys232, Glu260, Asp305, and Arg351 of the NACHT domain, while alantolactone interacts with Arg335 in a similar manner [123,124]. More recently, chloranthalactone B was reported to form a covalent bond between its epoxide group and Cys279 of the NLRP3 NACHT domain, thereby specifically disrupting the NEK7-NLRP3 interaction [126].

In contrast, some natural compounds act primarily by targeting the NEK7 protein itself. Licochalcone B has been shown to specifically bind NEK7, directly blocking its interaction with NLRP3 [125]. Anemoside B4 binds to NEK7 at Ser46 and Phe168, thereby disrupting the NEK7-NLRP3 interaction interface [118]. In addition, several other natural products, including theaflavin, gallic acid, and artemisinin, have been reported to inhibit the NEK7-NLRP3 interaction, although their precise binding sites remain unclear [122,127,130].

These findings collectively reveal the structural basis for how natural products suppress inflammasome activation at the molecular level, emphasizing their potential as inhibitors of the NEK7-NLRP3 interaction. The unique targeting profiles of these natural compounds, which either focus on NLRP3 or NEK7, offer valuable insights for the rational design of novel NLRP3 inflammasome modulators that exhibit improved selectivity and reduced toxicity.

#### 5.10.4 Targeting the adaptor protein ASC

ASC serves as the central adaptor protein in the inflammasome. Its PYD domain engages with NLRP3, while the CARD domain facilitates interaction with pro-caspase-1. This interaction triggers ASC speck formation and initiates inflammatory cascades. By blocking ASC oligomerization or its interaction with caspase-1, it is possible to prevent the assembly of a functional inflammasome complex. Notably, the natural compound caffeic acid phenethyl ester has been shown to bind directly to the PYD domain of ASC, effectively inhibiting ASC speck formation [129]. Similarly, rhein also exhibits an inhibitory effect on ASC speck formation [128]. In addition, coptisine and sennoside A suppress the cleavage and activation of pro-caspase-1 by disrupting its interaction with ASC [112,113].

## 6 Toxicology and adverse effects

Therapeutic effects and side effects are two inseparable aspects of all bioactive substances. Any bioactive compound with therapeutic potential can also have off-target effects on normal tissues. For example, resveratrol shows dose-dependent bidirectional activities: it demonstrates antioxidant properties at low doses, while exhibiting pro-oxidant effects at high doses [131]. In rat cardiotoxicity studies, administration of 100 mg/kg resveratrol resulted in 100% mortality due to severe cardiotoxicity [132]. Clinical trials have shown that a daily dose of 450 mg of resveratrol is safe for healthy individuals. However, for those with compromised health, high doses (>1000 mg/day) may increase cardiovascular risk markers, including oxidized low-density lipoprotein and soluble E-selectin-1 [133]. Moreover, resveratrol suffers from poor bioavailability, which limits its therapeutic efficacy [134]. A study reported that the median lethal dose (LD_50_) of sennoside A is approximately 5000 mg/kg in both rats and mice. This high LD_50_ value may be attributed to its laxative effect, which can result in dehydration and electrolyte imbalance [135]. Chronic toxicity studies have shown that kaempferol at a dose of 2000 mg/kg does not induce hepatotoxicity, nephrotoxicity, or hematotoxicity in mice [136]. Nevertheless, kaempferol exhibits cytotoxic effects against human tumor cells, suggesting its potential as an anti-cancer agent [137].

Subchronic toxicity studies in rats showed that a dose of 75 mg/kg of dioscin significantly increased urinary protein and creatinine levels in male rats, along with causing weight loss. When the dose was increased to 300 mg/kg, dioscin resulted in severe toxic effects, including hemolytic anemia and intestinal distension [138]. No significant toxicity was observed in female rats. Notably, clinical use of dioscin has been associated with adverse effects such as gastrointestinal discomfort, hepatotoxicity, and palpitations [139]. Furthermore, dioscin is limited by poor water solubility and low bioavailability [140]. Carvacrol also exhibits low bioavailability, as it is rapidly absorbed in the gastrointestinal tract and quickly excreted via urine [141]. Acute toxicity studies indicate that the oral LD_50_ of carvacrol in rats is 810 mg/kg. In contrast, the intravenous and intraperitoneal LD_50_ values in mice are significantly lower, measuring 80 mg/kg and 73.30 mg/kg, respectively [142,143]. In human blood cell assays, high concentrations (0.15 and 0.2 mg/mL) of carvacrol demonstrated cytotoxic effects [144]. Genetic toxicity studies further revealed that carvacrol induces DNA damage in a dose-dependent manner [145]. Studies on the acute toxicity of piperine have shown that its intravenous LD_50_ in mice is 15.1 mg/kg [146].

Studies have reported that celastrol exhibits cardiotoxicity. Specifically, administering a dose of 2 mg/kg for seven consecutive days can lead to significant cardiac damage in rats [147]. Moreover, celastrol may lead to hepatotoxicity, nephrotoxicity, and reproductive toxicity. This underscores the necessity for further evaluation of its potential risks [148]. Although some studies suggest that andrographolide may possess nephrotoxic and reproductive toxic effects, these findings remain controversial [149].

In summary, natural products often display potent biological activities along with potential toxicities, both of which deserve equal consideration. Their structurally diverse and evolutionarily derived characteristics, coupled with unique bioactivity profiles, render them invaluable sources for discovering new drug leads and bioactive molecules. However, attention must also be paid to their safety profiles and possible adverse effects. In GA models, natural products demonstrate anti-inflammatory effects through synergistic mechanisms that involve multiple pathways, targets, and components. Nonetheless, their potential side effects, particularly gastrointestinal disturbances and hepatorenal injuries, necessitate further investigation in well-designed animal studies and clinical trials. Despite their structural and functional advantages over synthetic compounds, challenges such as low bioavailability and non-specific distribution impede their precise targeting to GA-affected tissues. Thus, future research should focus on comprehensive toxicity profiling, structural optimization of natural compounds, and the development of responsive drug delivery systems. These strategies will enhance therapeutic efficacy while minimizing toxicity, ultimately providing a scientific foundation and practical pathway for the clinical translation of natural products.

## 7 Challenges and limitations

GA, as a representative metabolic inflammatory arthropathy, is among the most rapidly increasing types of arthritis worldwide [4]. Its hallmark features include persistent hyperuricemia leading to MSU crystal deposition, recurrent episodes of acute joint inflammation, and irreversible bone destruction, which can result in severe complications in advanced stages. Timely and effective intervention during the inflammatory progression phase is essential to prevent irreversible joint damage. While the pathological mechanisms underlying MSU crystal-induced inflammation are relatively well understood, the molecular regulation of inflammation resolution remains incompletely elucidated. Additionally, effective therapeutic strategies targeting crystal deposition, inflammatory responses, and tissue damage are still lacking. Consequently, the limited understanding of inflammation resolution, the constraints of current drug therapies, and the inadequacy of treatment strategies pose significant challenges in GA research today.

Natural products provide unique benefits in modulating the complex inflammatory network in GA. Their multi-component synergy, ability to regulate multiple targets, and generally low toxicity contribute to their effectiveness. In the context of NLRP3 inflammasome-mediated inflammation, natural products demonstrate particular value by targeting both the initiation (e.g., NF-κB/MAPK pathways) and activation (e.g., ROS production, mitochondrial dysfunction, lysosomal rupture, and ion flux disturbances) phases of NLRP3 inflammasome, effectively suppressing the inflammatory cascade. They have the potential to transform GA treatment from the traditional single-target anti-inflammatory paradigm to a new, multi-pathway coordinated therapeutic approach. Therefore, the key scientific challenge lies in quantitatively characterizing and fully harnessing this synergistic regulatory mechanism to overcome the limitations of traditional single-target interventions and off-target effects, ultimately leading to the development of multi-level, highly synergistic therapeutic strategies.

Numerous studies have demonstrated that various natural products can modulate GA by directly inhibiting specific NLRP3 domains or regulating their upstream signaling pathways. However, translating these findings into clinically viable formulations remains challenging. Most current evidence is derived from *in vitro* and animal studies, and the multi-pathway regulation of NLRP3 inflammasome activation by MSU crystals requires further validation in more human-relevant, complex microenvironments. Therefore, it is critical to develop *in vivo* GA models that simulate specific pathological perturbations to elucidate the mechanisms of action of natural products. Clinical trials are also indispensable for evaluating the safety and efficacy of new therapies. Thus, promoting well-designed clinical studies is imperative to assess the therapeutic effects, safety profile, and long-term benefits of natural products in GA patients. Furthermore, investigating combination therapies that incorporate natural products alongside existing agents for the treatment of GA, such as colchicine and IL-1 inhibitors, is essential for improving therapeutic efficacy and reducing adverse effects. Clinical evidence shows that monotherapy with urate-lowering or anti-inflammatory agents frequently falls short of achieving optimal results. Notably, certain natural products have demonstrated the ability to simultaneously regulate both uric acid levels and inflammatory responses. Thus, utilizing the multi-target properties of natural products to explore their dual modulation of inflammation and uric acid homeostasis presents a promising research avenue with considerable translational potential.

Currently, no US Food and Drug Administration (FDA) define-approved drugs specifically targeting the NLRP3 inflammasome have been developed, and most candidate compounds remain in the preclinical research phase. Natural products face inherent challenges such as low bioavailability, limited targeting precision toward inflammatory sites, potential off-target effects due to multi-target interactions, and difficulties in quality control. These limitations collectively hinder their clinical translation. Therefore, integrating modern technologies, such as nanodelivery systems and prodrug strategies, is essential to enhance the bioavailability and targeted delivery of bioactive natural compounds. The core of quality control for natural products lies in the integration of multi-dimensional analytical techniques and intelligent dynamic toxicity prediction models, enabling the establishment of a standardized quality control system from raw material cultivation to final product. A "chemistry-biology-intelligence" trinity paradigm, guided by biological activity, should be established to achieve a fundamental shift from empirical assessment to precision-based control.

Although natural products show considerable potential in targeting the NLRP3 inflammasome for the treatment of GA, current preclinical studies face multiple challenges that limit precise modulation of NLRP3 inflammasome. First, natural product-derived lead compounds often engage multiple signaling pathways during the initiation and activation of NLRP3 inflammasome, necessitating further structural optimization to enhance their specificity. Second, the complexity of the PTM network regulating the NLRP3 inflammasome represents a significant bottleneck. The regulation of NLRP3 inflammasome activity is highly context-dependent, influenced by cell type, microenvironment, and stimulation conditions, complicating the development of a unified regulatory model. Moreover, studies on PTMs regulating the NLRP3 inflammasome in GA models remain limited. Third, the functional necessity, synergy, and redundancy among NLRP3 inflammasome activation pathways, as well as their potential cross-talk mechanisms in GA models, remain poorly understood. Furthermore, the discovery of novel positive and negative regulatory factors is lagging, with current research heavily reliant on established regulators such as NEK7 and TXNIP. This reliance limits the diversity of natural product-based intervention strategies. To address these gaps, it is essential to employ comprehensive approaches, including multi-omics technologies, super-resolution microscopy, and patient-derived primary cell models, to systematically map the landscape of PTMs and the spatiotemporal regulatory networks of NLRP3 inflammasome in the context of GA. Such efforts will facilitate a deeper analysis of hierarchical regulatory relationships and cross-talk mechanisms. Additionally, research should aim to identify and validate novel NLRP3 inflammasome regulators with therapeutic potential in GA pathology through high-throughput screening and functional genomics.

Current research focus exhibits a significant imbalance. The majority of investigations concentrate on inhibiting NLRP3 inflammasome assembly or its priming and activation phases, while downstream effector mechanisms in GA, particularly pyroptosis execution, remain inadequately explored. Of particular note is the insufficient understanding of GSDMD's functional role in this context. Therefore, it is necessary to broaden the research perspective and investigate the specific mechanisms by which GSDMD contributes to GA-associated inflammation and tissue damage, and to systematically evaluate the therapeutic potential of targeting GSDMD. Additionally, whether natural products exert their effects through modulation of non-classical NLRP3 inflammasome activation pathways remains an understudied area. Thus, further clarification is needed regarding the involvement of candidate molecules in non-classical pathway regulation to improve preclinical safety and mechanism-based evaluation systems.

## 8 Conclusions and perspectives

In conclusion, the NLRP3 inflammasome is crucial to the inflammatory pathology of GA, and various natural products have shown therapeutic potential in reducing inflammation and joint tissue damage through different mechanisms. An ideal natural therapeutic agent for GA should effectively and sequentially target key regulatory nodes in the NLRP3 inflammasome activation cascade, including priming, PTMs, activation, assembly, and effector functions. This approach aims to minimize off-target effects and enhance therapeutic specificity. However, significant challenges persist in fully understanding the mechanisms by which MSU crystals activate the NLRP3 inflammasome. Additionally, optimizing the screening and structural modification of bioactive natural compounds, improving their bioavailability, and facilitating clinical translation remain critical tasks. In summary, targeting the NLRP3 inflammasome with natural products is a promising and scientifically valuable strategy for treating GA, which warrants further in-depth investigation.

## CRediT authorship contribution statement

**Yunhao Yi:** Writing – original draft. **Yanling Chen:** Writing – review & editing. **Wuchaonan Liu:** Writing – review & editing. **Jingjing Yang:** Writing – review & editing. **Le Yang:** Writing – review & editing. **Jing Liu:** Writing – review & editing. **Shengping Luo:** Writing – review & editing. **Qianru Zeng:** Writing – review & editing. **Tao Gao:** Writing – review & editing. **Yihui Deng:** Writing – review & editing.

## Declaration of generative AI and AI-assisted technologies in the manuscript preparation process

During the preparation of this work, the authors used ChatGPT (OpenAI) in order to assist with initial translation and grammar editing. The tool was not used to generate scientific content, analyses, or conclusions. After using this tool, the authors reviewed and edited the content as needed and take full responsibility for the content of the published article.

## Declaration of competing interest

The authors declare that they have no known competing financial interests or personal relationships that could have appeared to influence the work reported in this paper.
